# Magnetic Structures and Magnetic Phase Diagram of the Mixed-Valence Iron Phosphate Fe_7_(PO_4_)_6_

**DOI:** 10.1021/acs.inorgchem.6c00534

**Published:** 2026-03-26

**Authors:** Andreas Dönni, Lukas Keller, Vladimir Y. Pomjakushin, Naohito Tsujii, Alexei A. Belik

**Affiliations:** † Research Center for Materials Nanoarchitectonics (MANA), 52747 National Institute for Materials Science (NIMS), Namiki 1-1, Tsukuba, Ibaraki 305-0044, Japan; ‡ PSI Center for Neutron and Muon Sciences, Villigen CH-5232, Switzerland

## Abstract

Mixed-valence iron compounds are fascinating materials exemplified by ferrimagnetic Fe_3_O_4_, multiferroic LuFe_2_O_4_, and a pigment Fe_4_[Fe­(CN)_6_]_3_. Mixed-valent iron (oxy)­phosphates also provide a large variety of different connections between magnetic ions, which are responsible for complex magnetism. Fe_7_(PO_4_)_6_ (Fe_3_^2+^Fe_4_^3+^(PO_4_)_6_) has well-defined Fe^2+^ and Fe^3+^ sites and shows two antiferromagnetic transitions at *T*_N1_ = 47 K and *T*_N2_ = 16 K. Here, we investigated magnetic structures of Fe_7_(PO_4_)_6_ (at zero magnetic field) using neutron powder diffraction and constructed a temperature–magnetic-field phase diagram. Below *T*_N1_, magnetic propagation vector is ***k***_**1**_ = (1/2, 0, 1/2), and magnetic moments on Fe^3+^ sites are nearly fully ordered; while moments on Fe^2+^ sites are significantly reduced. Below *T*_N2_, the second propagation vector appears ***k***_**2**_ = (0, 1/2, 0) and coexists with ***k***_**1**_; moments on Fe^2+^ sites are fully ordered (at 2 K) reaching 4.5 μ_B_ suggesting noticeable spin–orbital contributions. Coexistence of two propagation vectors and triclinic symmetry results in variations of total magnetic moments on each site. Magnetic measurements up to 300 kOe detected one field-induced transition near 55.5 kOe. Data showed that *T*_N1_ is nearly magnetic-field independent up to 90 kOe, while a more complex behavior was observed near *T*_N2_ at magnetic fields around 50 kOe.

## Graphical abstract

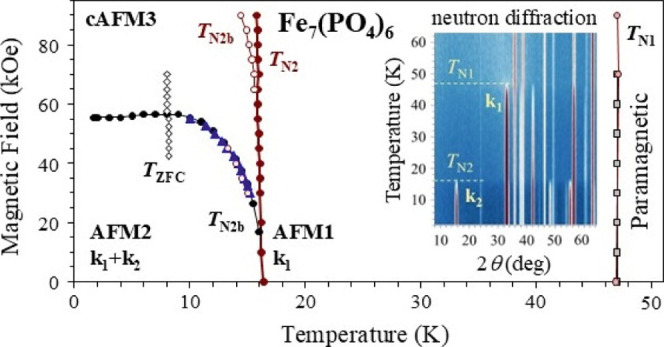

## 1 Introduction

Mixed-valence (MV) compounds contain elements which are present in more than one (formal) oxidation state, usually in two oxidation states.^1–3^ MV compounds are important in biology, where they are responsible for photosynthesis and respiration (a Fe^2+^/Fe^3+^ pair), physics, and chemistry.^4^ In inorganic chemistry, mixed valency can produce very important properties. For example, a deep blue widely used pigment Fe_4_[Fe­(CN)_6_]_3_ is a MV compound,^5^ high-*T*_C_ copper superconductors are often MV compounds.^6^ MV compounds are at the core of batteries.^7^ MV manganites produce ferromagnetism and giant magnetoresistance properties.^3,8^ Interestingly, in manganites prepared at high pressure, it is suggested that Mn can even be in three oxidation state of +2, +3, and +4, for example, in a perovskite-like Mn_2_O_3_,^9^ (R_1–*x*_Mn_*x*_)­MnO_3_,^10^ and RMn_3_O_6_,^11^ where R is a rare-earth element. The MV character of some minerals provides the basis for their color. Mixed valency is often a prerequisite for high electrical conductivity in nonmetallic materials.

Different phosphates and MV iron phosphates and oxyphosphates also provide a large variety of different connections between magnetic ions, which are responsible for complex magnetism.^12,13^ The MV iron phosphate Fe_7_(PO_4_)_6_ (=Fe_3_^2+^Fe_4_^3+^(PO_4_)_6_) was first discovered in 1980.^14^ It crystallizes in the triclinic space group *P*1̅ (no. 2) and magnetic iron ions (Fe1^2+^, Fe2^2+^, Fe3^3+^, Fe4^3+^) are located on four different sites.^14^ The crystal structure is illustrated in Figure 1. The environment of the Fe^2+^ ions is octahedral Fe1O_6_ and pyramidal Fe2O_5_. The ferric Fe^3+^ ions have octahedral Fe3O_6_ and Fe4O_6_ coordination. The Fe1O_6_, Fe2O_5_, Fe3O_6_ and Fe4O_6_ polyhedra form a three-dimensional network. There are zigzag chains propagating along (0, 1, −1) directions formed by edge-shared polyhedra, ···–Fe3O_6_–Fe3O_6_–Fe2O_5_–Fe4O_6_–Fe4O_6_–Fe2O_5_–Fe3O_6_–···(Figure 1). These chains are linked with each other through the Fe1O_6_ polyhedra by corner-shared connections Fe1O_6_–Fe2O_5_ and Fe1O_6_–Fe3O_6_. In addition, the Fe^3+^ ions, Fe3^3+^ and Fe4^3+^, each form dimer units through the edge-shared polyhedra Fe3O_6_–Fe3O_6_ as well as Fe4O_6_–Fe4O_6_.

**Figure 1:**
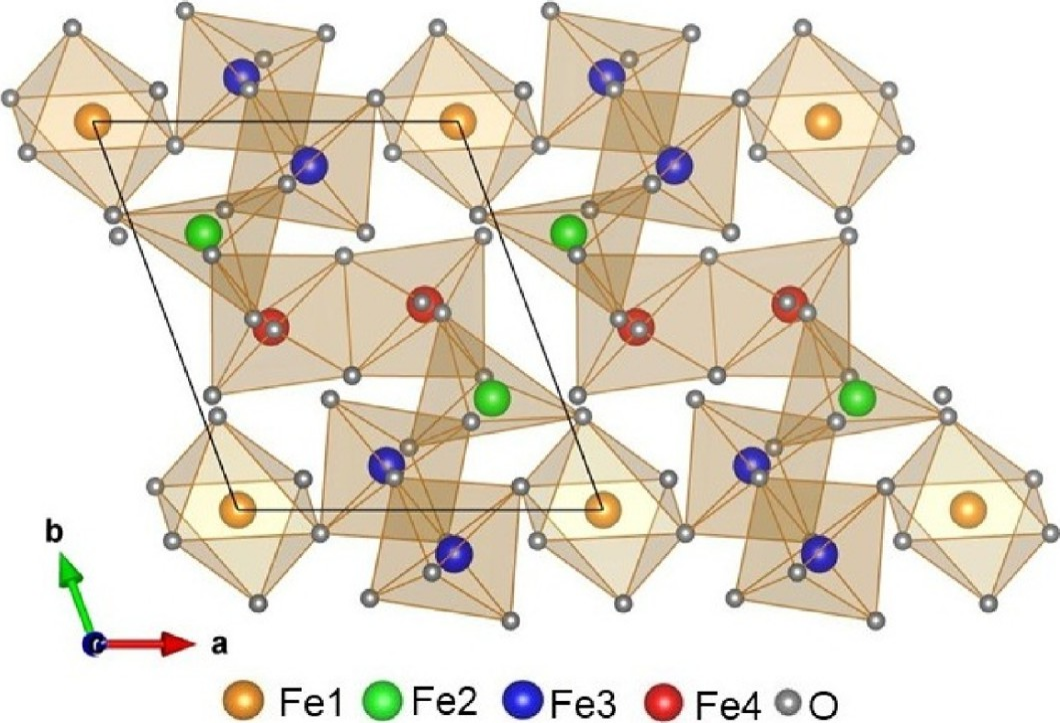
Triclinic crystal structure of Fe_7_(PO_4_)_6_, where only FeO_n_ polyhedra are shown and the phosphor (P) ions are omitted. The drawing was made using VESTA software.^15^

Some physical properties of Fe_7_(PO_4_)_6_ were investigated later.^16–19^ Even though it has well-defined Fe^2+^ and Fe^3+^ sites from the structural analysis and Mössbauer spectroscopy and shows insulating properties, it has a deep black color similar to MV iron minerals, magnetite, Fe_3_O_4_,^20^ and ilvaite, Ca­(Fe^2+^,Fe^3+^)­Fe^3+^Si_2_O_7_O­(OH),^21^ suggesting some degree of electron transfer.

The crystal structure type of Fe_7_(PO_4_)_6_ is quite adaptive as other phosphates,^22–27^ some vanadates (e.g., Cu_3_Fe_4_(VO_4_)_6_, NaCuFe_2_(VO_4_)_3_, LiCuFe_2_(VO_4_)_3_),^28–32^ molybdates (e.g., Na_2_Zn_5_(MoO_4_)_6_),^33^ and arsenates (e.g., Fe_7_(AsO_4_)_6_ and Mn_7_H_4_(AsO_4_)_6_)^34–36^ can crystallize in the same structure type. The number of cations can vary from about 6.5 to 8 per formular unit allowing, for example, applications in batteries^27^ and Na-ion intercalation.^18^ Some phosphates are colorful pigments,^26^ and LiCuFe_2_(VO_4_)_3_ shows multiferroic properties.^32^ The structure can also contain hydrogen in the form of O–H groups, for example, in Co_7_H_4_(PO_4_)_6_,^37^ Mn_7_H_4_(PO_4_)_6_,^36^ Mn_7_H_4_(AsO_4_)_6_,^36^ Fe_7_H­(PO_4_)_6_,^38,39^ and Mg_7_H_4_(PO_4_)_6_.^40^

In this work, we investigated the magnetic structures of the parent compound Fe_7_(PO_4_)_6_ using neutron powder diffraction. Fe_7_(PO_4_)_6_ shows two successive antiferromagnetic (AFM) transitions at *T*_N1_ = 47 K and *T*_N2_ = 16 K.^18,19^ The magnetic propagation vector ***k***_**1**_ = (1/2, 0, 1/2) appears below *T*_N1_. There is a noncollinear AFM structure, where all ordered moments (of the ***k***_**1**_ component) lie inside one plane that rotates around the *c*-direction near *T*_N2_. At *T* = 25 K, the ordered moments are large on the Fe^3+^ sites (about 4 μ_B_) and significantly reduced on the Fe^2+^ sites (less than 2 μ_B_). Below *T*_N2_, the magnetic structure has a second magnetic propagation vector ***k***_**2**_ = (0, 1/2, 0) that coexists with ***k***_**1**_. Below *T*_N2_, magnetic moments on the Fe^2+^ sites consist of a large ***k***_**2**_ and a small ***k***_**1**_ component, whereas the moments on the Fe^3+^ sites have a large ***k***_**1**_ and a small ***k***_**2**_ component. At the ground state, the coexistence of two propagation vectors (with ***k***_**1**_ and ***k***_**2**_ components predominantly perpendicular to each other) and the triclinic crystal symmetry results in variations of total magnetic moments on each site. At the lowest measured temperature of 2 K, all Fe moments are fully ordered. Magnetic moments on the Fe^2+^ sites reach about 4.5 μ_B_—larger than the spin-only value of 4.0 μ_B_—suggesting noticeable spin–orbital contributions. We also constructed a temperature-magnetic field phase diagram using temperature-dependent and field-dependent magnetization and specific heat measurements and measured magnetization up to 300 kOe.

## 2 Experimental Section

Single-phase black Fe_7_(PO_4_)_6_ was synthesized by a standard solid-state method from a stoichiometric mixture of FePO_4_ and Fe (99.9%) by annealing at 1173 K for 130 h as a pellet in an evacuated sealed quartz tube (to prevent the oxidation of Fe^2+^) with several intermediate grindings. Single-phase yellow FePO_4_ was prepared by a standard solid-state method from a stoichiometric mixture of Fe_2_O_3_ (99.999%) and NH_4_H_2_PO_4_ (99.9%) by annealing at 1073 K for 60 h in air with several intermediate grindings. Phase purity of the compounds was confirmed through X-ray powder diffraction measurements using an Ultima-IV Rigaku diffractometer (with Cu Kα radiation).

A large amount of Fe_7_(PO_4_)_6_ sample (about 6 g) was used to perform powder neutron diffraction experiments at the Paul Scherrer Institute, Switzerland. The sample was mounted in a cylindrical vanadium­(V) can and placed in a helium cryostat for temperature-dependent measurements. The crystal structure was measured in the paramagnetic state at *T* = 60 K on the high-resolution powder diffractometer for thermal neutrons (HRPT)^41^ using an incident neutron wavelength of λ = 1.886 Å. Data were collected for a 2θ range of 3.55°–164.50° and a step width of 0.05°. Data for the magnetic structure analysis were measured on the cold neutron diffractometer DMC using an incident neutron wavelength of λ = 4.507 Å. Data were collected at 2, 25, and 60 K for a 2θ range of 5.0°–137.9° and a step width of 0.1°. The temperature dependence was measured between 2 and 80 K for cooling and heating cycles by ramping at a constant rate of 0.2 K/min. Neutron diffraction data were continuously collected throughout the temperature ramps, with data files written at 1 min intervals.

The diffraction patterns were analyzed by the Rietveld method using the FullProf Suite.^42^ Possible models for the magnetic structures were deducted based on a group theory analysis using the programs ISODISTORT^43,44^ and BASIREPS in the FullProf Suite program package.^42^

Magnetic properties were measured on a SQUID magnetometer (Quantum Design MPMS3, San Diego, CA, USA) in different applied fields under both zero-field-cooled (ZFC) and field-cooled on cooling (FCC) conditions. Magnetic field dependence of magnetization was measured at different temperatures between −70 and 70 kOe (or between 0 and 70 kOe). Isothermal magnetization curves were also taken at 1.7 K between 0 and 300 kOe using a (former) hybrid magnet of National Institute for Materials Science (NIMS, Tsukuba, Japan). Specific heat, *C*_p_, was measured during cooling at different magnetic fields using a pulse relaxation method with a commercial calorimeter (Quantum Design PPMS, San Diego, CA, USA).

## 3 Results and Discussion

### 3.1 Crystal Structure of Fe_7_(PO_4_)_6_

The crystal structure of Fe_7_(PO_4_)_6_ at room temperature has been reported based on single crystal^14^ and powder X-ray diffraction.^16^ We have determined the structural parameters of paramagnetic Fe_7_(PO_4_)_6_ at *T* = 60 K by neutron diffraction. The refinement is shown in Figure 2 and the results are summarized in Table 1. Calculated values for selected bond lengths and bond angles are given in Table S1 (Fe–Fe bond lengths, Fe–O–Fe bond angles), Table S2 (Fe–O bond lengths) and Table S3 (P–O bond lengths and O–P–O bond angles). During the refinement, atomic displacement parameters were constrained to be the same for all Fe sites, all P sites, and all O sites. Bond-valence sum values (the note of Table 1)^45^ support the oxidation state of +2 for the Fe1 and Fe2 sites, and +3 for the Fe3 and Fe4 sites. The network of the magnetic Fe ions in the crystal structure is illustrated in Figure 3 with Fe–Fe bond lengths indicated up to a distance of 3.6 Å below which the neighboring Fe–O polyhedra all share a common edge or corner. As shown in Figure 3, the shortest Fe–Fe distances all appear inside the zigzag chains···–Fe2′–Fe3′–Fe3–Fe2–Fe4–Fe4′–Fe2′–···with edge-shared Fe–O polyhedra. Fe*i*′ (*i* = 2, 3, 4) is connected to Fe*i* at (*x*, *y*, *z*) by inversion to (*x̅*, *y̅*, *z̅*) and a translation vector (*t*_*x*_, *t*_*y*_, *t*_*z*_) of lattice constants. Zigzag chains propagate along (0, 1, −1) directions. For Fe1, Fe–Fe bond lengths to the four nearest neighbors Fe2, Fe2′, Fe3 and Fe3′ with corner shared Fe–O polyhedra are almost constant. The four nearest neighbors belong to four different zigzag chains.

**Figure 2:**
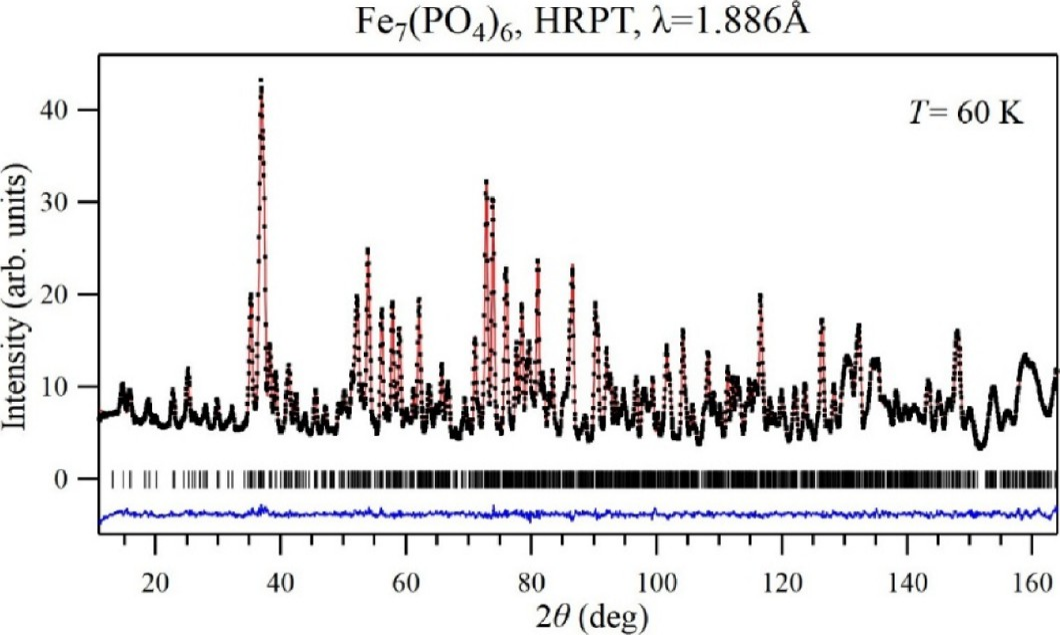
Experimental (black dots), calculated (red line), and difference (blue line) neutron diffraction patterns of Fe_7_(PO_4_)_6_ measured on HRPT with a neutron wavelength λ = 1.886 Å in the paramagnetic state at *T* = 60 K. Tick marks indicate Bragg peak positions.

**Table 1:** Refined Structural Parameters of Fe_7_(PO_4_)_6_ Phosphate in the Paramagnetic State Obtained from Rietveld Refinement Analysis of HRPT Neutron Diffraction Data Measured at *T* = 60 K with λ = 1.886 Å^a^ ^b^ ^c^

atom	WP	*x*/*a*	*y*/*b*	*z*/*c*	*B* (Å^2^)
Fe1	1*a*	0	0	0	0.12(2)
Fe2	2*i*	0.8108(2)	0.2876(2)	0.2810(3)	0.12(2)
Fe3	2*i*	0.4527(2)	0.1141(2)	0.3828(3)	0.12(2)
Fe4	2*i*	0.7226(2)	0.5291(2)	0.0443(3)	0.12(2)
P1	2*i*	0.5938(4)	0.8331(4)	0.0965(5)	0.10(3)
P2	2*i*	0.2318(4)	0.3706(4)	0.3988(5)	0.10(3)
P3	2*i*	0.1510(4)	0.7667(4)	0.2285(5)	0.10(3)
O1	2*i*	0.0398(3)	0.2411(3)	0.2753(4)	0.28(2)
O2	2*i*	0.5393(3)	0.9170(3)	0.3120(5)	0.28(2)
O3	2*i*	0.2835(4)	0.4651(3)	0.2540(5)	0.28(2)
O4	2*i*	0.3661(4)	0.2855(3)	0.4494(5)	0.28(2)
O5	2*i*	0.2709(4)	0.7728(3)	0.4575(5)	0.28(2)
O6	2*i*	0.5501(4)	0.6539(3)	0.0665(5)	0.28(2)
O7	2*i*	0.7891(4)	0.9203(3)	0.1199(5)	0.28(2)
O8	2*i*	0.5321(3)	0.1609(3)	0.1202(5)	0.28(2)
O9	2*i*	0.8105(4)	0.3425(3)	0.9808(5)	0.28(2)
O10	2*i*	0.7608(4)	0.5073(3)	0.3664(5)	0.28(2)
O11	2*i*	0.2018(3)	0.9397(3)	0.2257(5)	0.28(2)
O12	2*i*	0.9528(4)	0.7054(3)	0.2110(5)	0.28(2)

a Space group *P*1̅ (No. 2); *Z* = 1. WP: Wyckoff position. *B*: Debye Waller factor. The oxidation states of the magnetic ions are Fe1^2+^, Fe2^2+^, Fe3^3+^ and Fe4^3+^.

b Lattice parameters and unit cell volume: *a* = 7.9638(1) Å; *b* = 9.3073(1) Å; *c* = 6.3539(1) Å; α = 108.340(1)°; β = 101.628(1)°; γ = 105.213(1)°; *V* = 410.09(1) Å^3^. Bond-valence sum values^45^ are +1.86 for Fe1, +2.00 for Fe2, +3.08 for Fe3, and +3.05 for Fe4.

c *R*-factors: *R*_wp_ = 2.26%; *R*_exp_ = 1.62%; *R*_Bragg_ = 1.18%; χ^2^ = 1.94.

**Figure 3:**
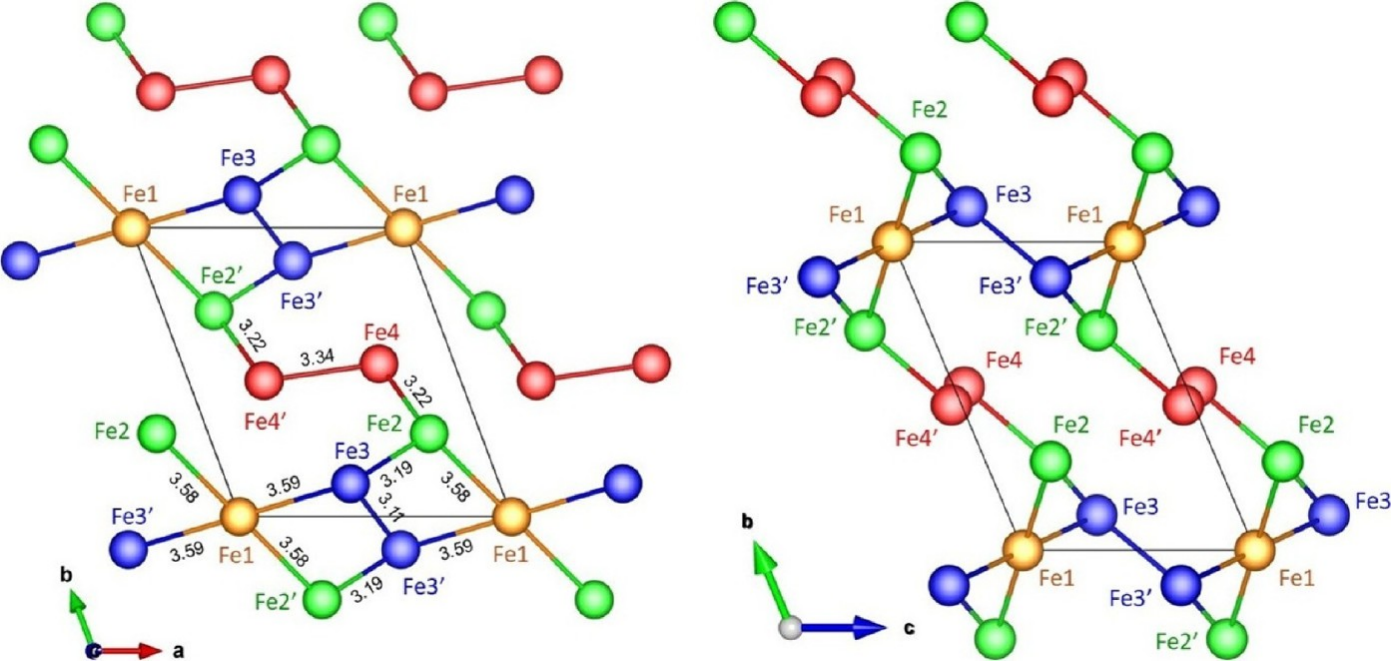
Arrangement of magnetic Fe ions in the crystal structure of Fe_7_(PO_4_)_6_ shown as a projection onto the *ab*- and *bc-*plane. Fe–Fe distances up to 3.6 Å are indicated. Drawings were made using VESTA software.^15^

### 3.2 Magnetic Phase Transitions and Magnetic Phase Diagram of Fe_7_(PO_4_)_6_

The temperature dependence of *C*_p_ and *C*_p_/*T* data at *H* = 0 Oe is shown on Figure 4. We observed strong and sharp λ-type anomalies at *T*_N1_ = 47 K and *T*_N2_ = 16 K in agreement with the previous reports.^18,19^ The *C*_p_ and *C*_p_/*T* data at *H* = 90 kOe showed that *T*_N1_ is nearly magnetic-field independent up to 90 kOe. On the other hand, a clear double-peak anomaly was observed near *T*_N2_, when measured with a fine measurement step of 0.1 K. Differential d*C*_p_/d*T* versus *T* curves clearly demonstrated peaks at 14.4 and 15.8 K at *H* = 90 kOe (Figures 4 and S1, Table S4). The double-peak features (with both down-peaks on the d*C*_p_/d*T* versus *T* curves) remained from 90 kOe down to 65 kOe and disappeared from 60 kOe down to 50 kOe. It is interesting that different features appeared from 45 kOe down to 35 kOe, where there was one weak up-peak on the d*C*_p_/d*T* versus *T* curves and one main strong down-peak (Figures S1 and S2). One peak on the d*C*_p_/d*T* versus *T* curves near 16 K is nearly magnetic-field independent up to 90 kOe, the second peak (which we call *T*_N2b_) showed slight field dependence. With the absence of available compounds with nonmagnetic elements, it was difficult to correctly estimate the lattice contribution and magnetic entropy. The *C*_p_/*T* versus *T* ^2^ curve (at *H* = 0 Oe) did not follow a linear behavior even at low temperatures; on the other hand, the following relation was approximately observed: *C*_p_ = β × *T* ^5^ with β = 2.792(6)×10^–4^ J × mol^–1^× K^–6^ between 1.9 and 6 K (Figures S3–S5). There were no detectable electronic contributions in agreement with the insulating properties.

**Figure 4:**
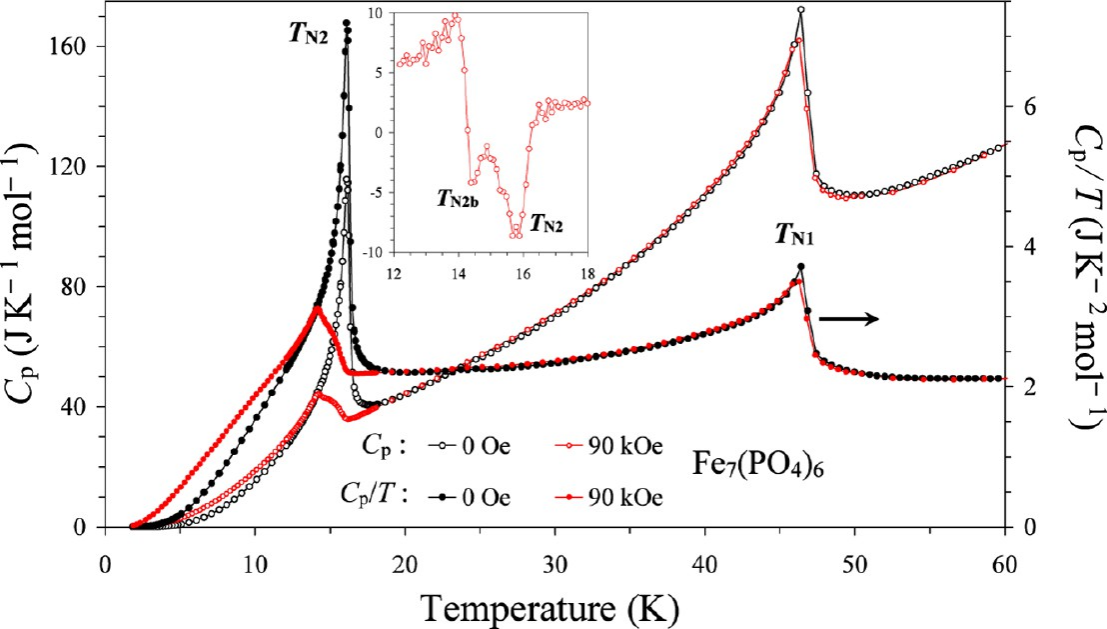
Specific heat, *C*_p_ versus *T* (the left-hand axis), and *C*_p_/*T* versus *T* curves (the right-hand axis) at *H* = 0 Oe (black) and 90 kOe (red) for Fe_7_(PO_4_)_6_, measured on cooling. Inset shows d*C*_p_/d*T* versus *T* curve at *H* = 90 kOe (units: J K^–2^ mol^–1^ versus K).

Isothermal *M* versus *H* curves clearly revealed the presence of one field-induced transition between 1.7 and 16 K (=*T*_N2_) (Figures S6 and S7, Table S5). The values of transition fields were determined from the differential d*M*/d*H* versus *H* curves measured from 70 kOe to 0 Oe; there was small hysteresis during measurements from 0 Oe to 70 kOe and from 70 kOe to 0 Oe (the inset of Figure 5). The transition field reaches maximum of 56.5 kOe (between 6 and 9 K) and then slightly decreases to 55.5 kOe (between 1.7 and 4 K). We also performed high-magnetic field *M* versus *H* measurements up to *H* = 300 kOe at *T* = 1.7 K (Figure 5). However, no additional magnetic field-induced transitions were observed. Magnetization reached about 13.9 μ_B_/f.u. (at *H* = 300 kOe and *T* = 1.7 K). The *M* versus *H* curves were linear up to 55 kOe, suggesting a pure AFM state, then gradually increased up to 300 kOe. The *M* versus *H* curves were fitted by a linear function between 250 kOe and 300 kOe and then extrapolated to zero magnetic field resulting in 2.85 μ_B_/f.u. (Figure 5, the blue thin line). This value can be considered as an induced moment due to spin canting in a canted antiferromagnetic (cAFM) state. The magnetic-field-temperature points from the *M* versus *H* curves match with *T*_N2b_(*H*, *T*).

**Figure 5:**
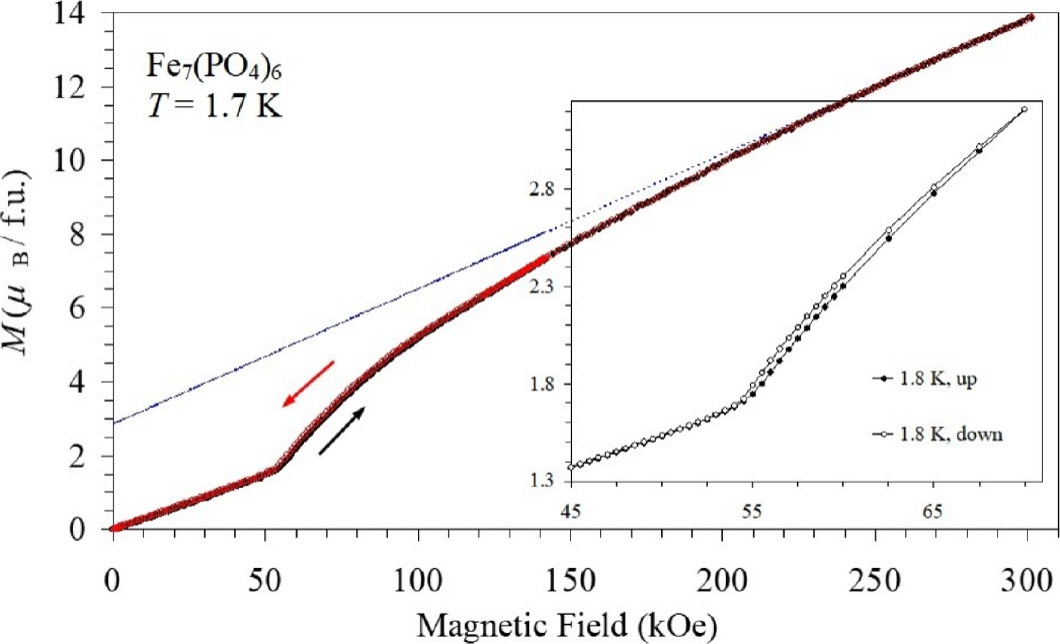
*M* versus *H* curves at *T* = 1.7 K from 0 Oe to 300 kOe (black) and from 300 kOe to 0 Oe (red) for Fe_7_(PO_4_)_6_, measured using a hybrid magnet. Inset shows a zoomed-in part of *M* versus *H* curves at *T* = 1.8 K, measured using MPMS3, and emphasizes a small hysteresis. A blue thin line shows a linear fit between 250 kOe and 300 kOe, which is then extrapolated to zero magnetic field.

Temperature-dependent susceptibility measurements, χ versus *T*, at *H* = 1, 10, 20, 30, 40, 50, 60, 70 kOe showed that *T*_N1_ is nearly magnetic-field independent in agreement with the specific heat measurements. Therefore, we focus on the behavior near *T*_N2_ (Figures 6 and S8–S13, Table S6). The χ versus *T* curves at all magnetic fields showed maxima at nearly the same temperature of 16 K, which appears as peaks on the double differential curves, d^2^ *χT*/d^2^ *T* versus *T*; these anomalies coincide with the specific heat anomalies. Above about 30 kOe up to 55 kOe, other sharp peaks start emerging on the differential curves, d*χT*/d*T* versus *T*, which match with anomalies on the d*M*/d*H* versus *H* curves and with *T*_N2b_. Between 42.5 kOe up to the maximum measurement field of 70 kOe, ZFC curves showed other sharp peaks on the differential curves, d*χT*/d*T* versus *T* (Figure 6), while no anomalies were observed on FCC curves. This transition is marked as *T*_ZFC_ (=8 K). Very small difference between ZFC and FCC curves was also observed above 42.5 kOe, and the difference became clear above 55 kOe. No difference between ZFC and FCC curves was detected below 40 kOe. The Curie–Weiss fit was performed between 200 and 350 K (using data measured at *H* = 50 kOe and 76.52 mg of the sample weight to increase accuracy); the Curie–Weiss temperature was θ = −68.92(14) K, and the experimental effective magnetic moment of μ_eff_ = 14.771(3) μ_B_ was close to the calculated value of μ_eff_ = 14.56 μ_B_ (for the spin-only values of 4Fe^3+^ and 3Fe^2+^).

**Figure 6:**
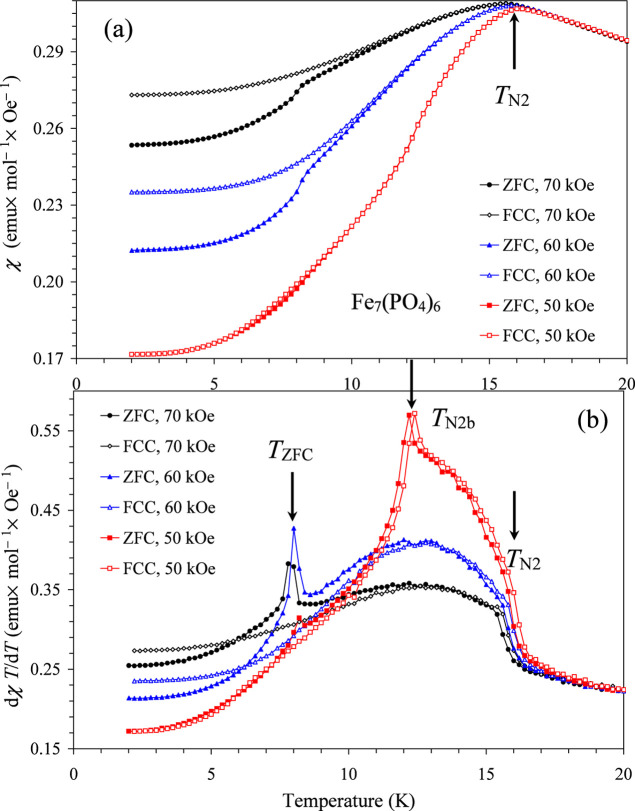
(a) χ versus *T* curves at *H* = 70 kOe (black), 60 kOe (blue), and 50 kOe (red) measured in the ZFC and FCC (on cooling) regimes for Fe_7_(PO_4_)_6_. (b) The same differential dχ*T*/d*T* versus *T* curves. Arrows show the magnetic anomalies.

Based on the results of the above measurements, the *H*–*T* phase diagram can be constructed for Fe_7_(PO_4_)_6_ (Figure 7). The appearance of a line only on the ZFC curves at *T*_ZFC_ = 8 K above 42.5 kOe may suggest the formation of metastable magnetic phases. The phase diagram has a triple point at *T*_N2_ and a magnetic field between 0 Oe and about 20 kOe; with the accuracy of measurements, it was not possible to determine the precise value of a magnetic field. There is evidence for the presence of another triple point at *T*_N2_ and a magnetic field of about 65 kOe.

**Figure 7:**
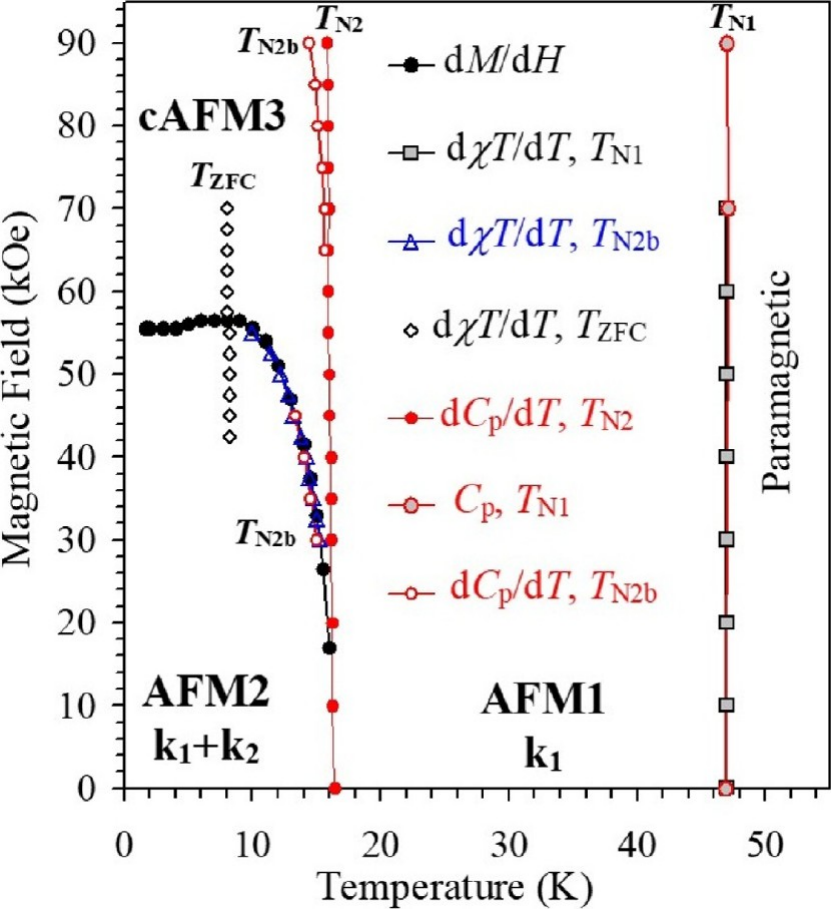
A *H* versus *T* phase diagram of polycrystalline Fe_7_(PO_4_)_6_. Experimental points were obtained from different measurements as indicated on the figure. The points from d^2^χ*T*/d^2^ *T* versus *T* curves, which define *T*_N2_, coincide with the points on the d*C*_p_/d*T* versus *T* curves (for *T*_N2_) and are not shown. AFM: antiferromagnetic; cAFM: canted antiferromagnetic.

### 3.3 Magnetic Structures of Fe_7_(PO_4_)_6_ at Zero Magnetic Field

Figure 8 shows the low angle part (10° < 2θ < 80°) of the refinement of neutron diffraction patterns of Fe_7_(PO_4_)_6_ measured with a large neutron wavelength λ = 4.507 Å in the paramagnetic state at (a) *T* = 60 K, and in the magnetically ordered states at (b) 25 K (between *T*_N1_ and *T*_N2_) and 2 K (below *T*_N2_). Simultaneous refinements of crystal and magnetic structures were performed in the full range of scattering angles 2θ up to 137.9° by keeping the atomic positions fixed at the values given in Table 1. The refinements over the full 2θ range are shown in Figure S14. At *T* = 25 K, all observed magnetic Bragg peaks can be indexed with a commensurate AFM propagation vector ***k***_**1**_ = (1/2, 0, 1/2). At 2 K, the propagation vector ***k***_**1**_ remains and a second propagation vector ***k***_**2**_ = (0, 1/2, 0) appears. The observed temperature dependence of selected magnetic Bragg peaks is displayed in Figures 9 and 10. Bragg peaks corresponding to ***k***_**1**_ appear below *T*_N1_ = 47 K and change intensity near *T*_N2_ = 16 K, whereas the magnetic Bragg peaks corresponding to ***k***_**2**_ are observed below *T*_N2_. The intensity map between 2 and 80 K is shown in Figure 9 for the heating cycle and in Figure S15 for the cooling cycle. Both cycles show identical intensities.

**Figure 8:**
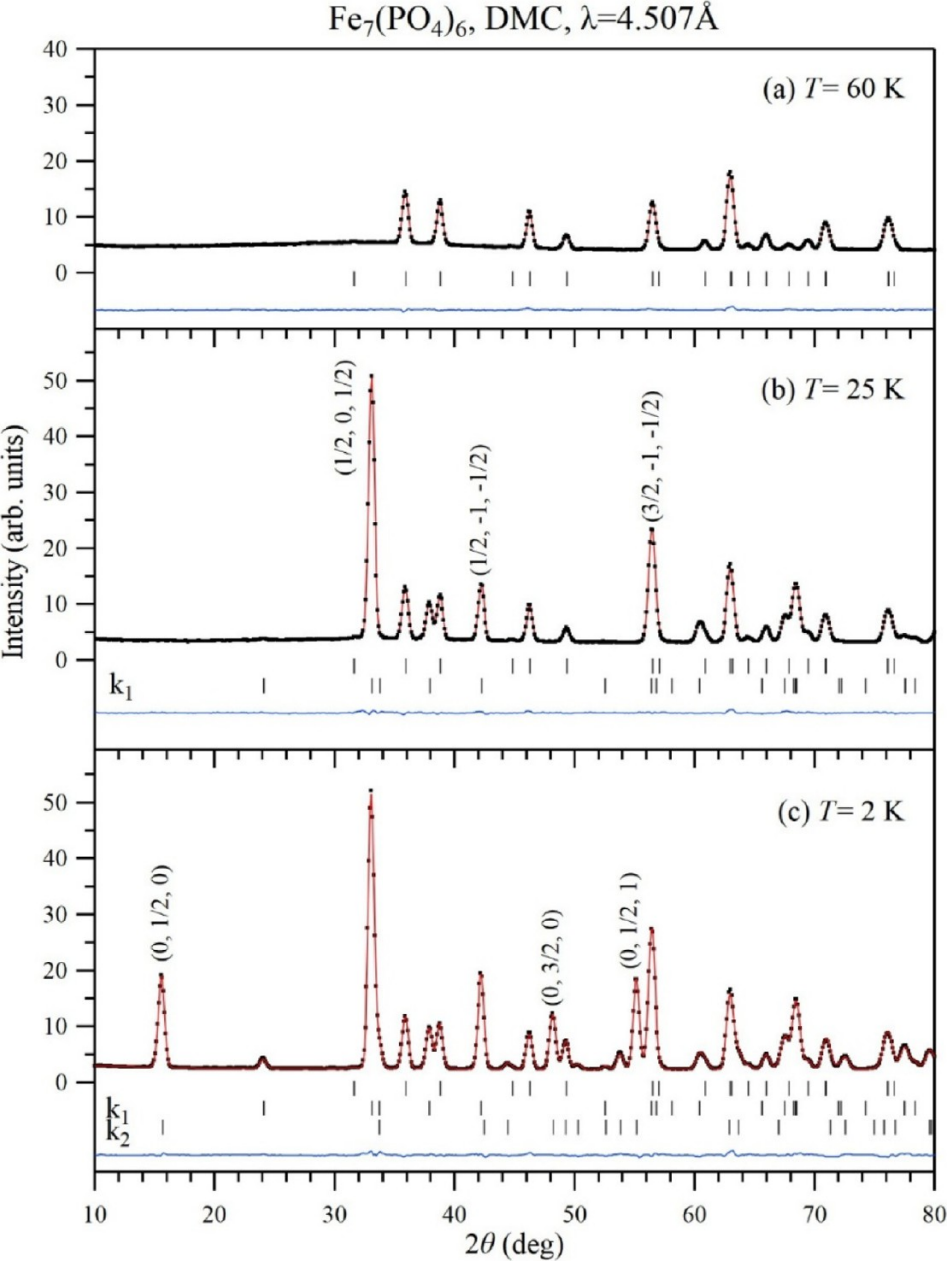
Experimental (black dots), calculated (red line), and difference (blue line) neutron diffraction patterns of Fe_7_(PO_4_)_6_ measured on DMC with a neutron wavelength λ = 4.507 Å in the paramagnetic state at *T* = 60 K (a) and in the magnetically ordered states at *T* = 25 K (b), and 2 K (c). Tick marks indicate Bragg peak positions. The first row is for the nuclear peaks, and the second and third rows are for the magnetic peaks. The three strongest magnetic Bragg peaks are indexed for ***k***_**1**_ at *T* = 25 K and for ***k***_**2**_ at *T* = 2 K.

**Figure 9:**
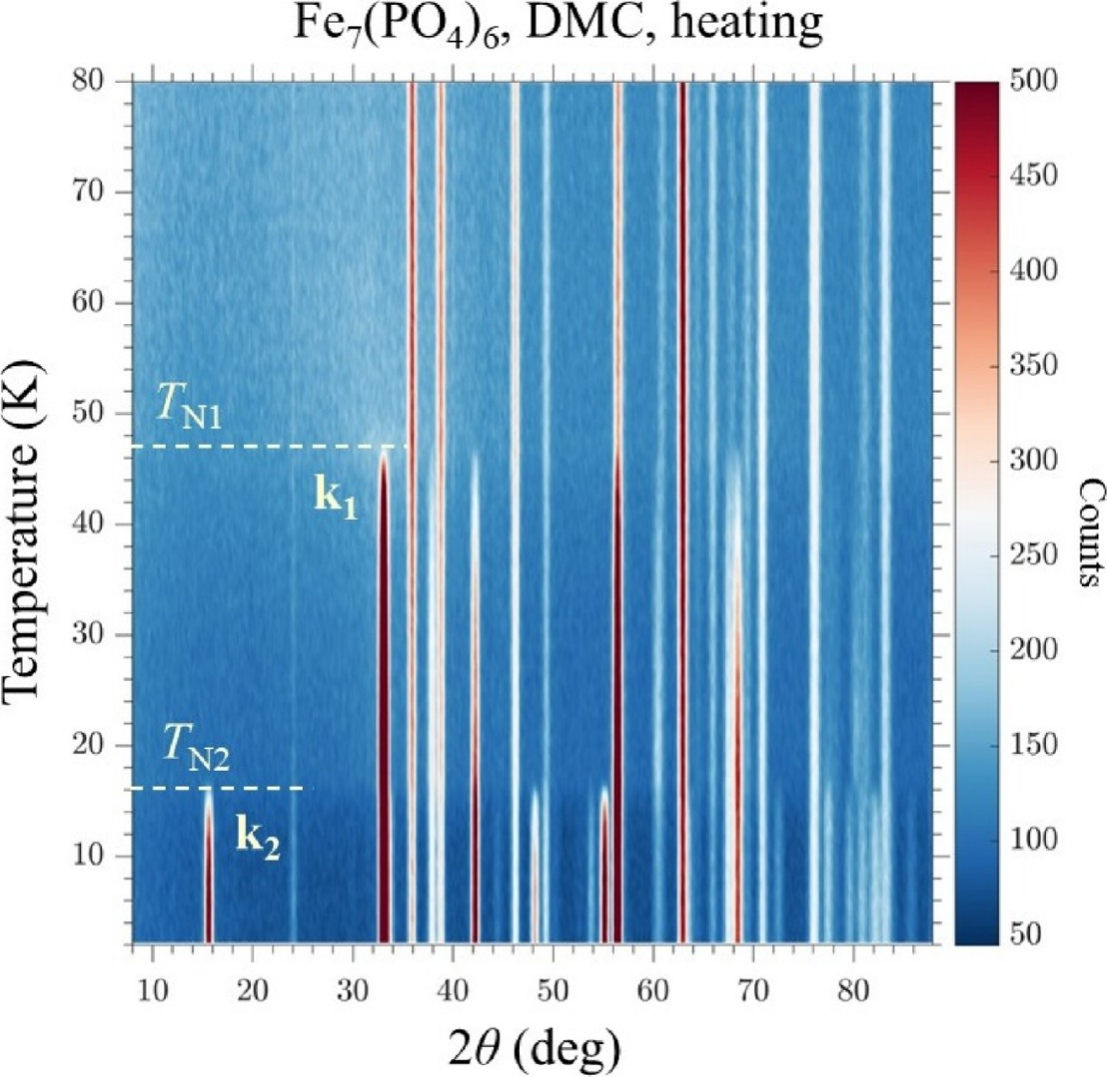
Temperature and 2θ dependent neutron intensity map of Fe_7_(PO_4_)_6_ measured on DMC for heating from 2 K.

**Figure 10:**
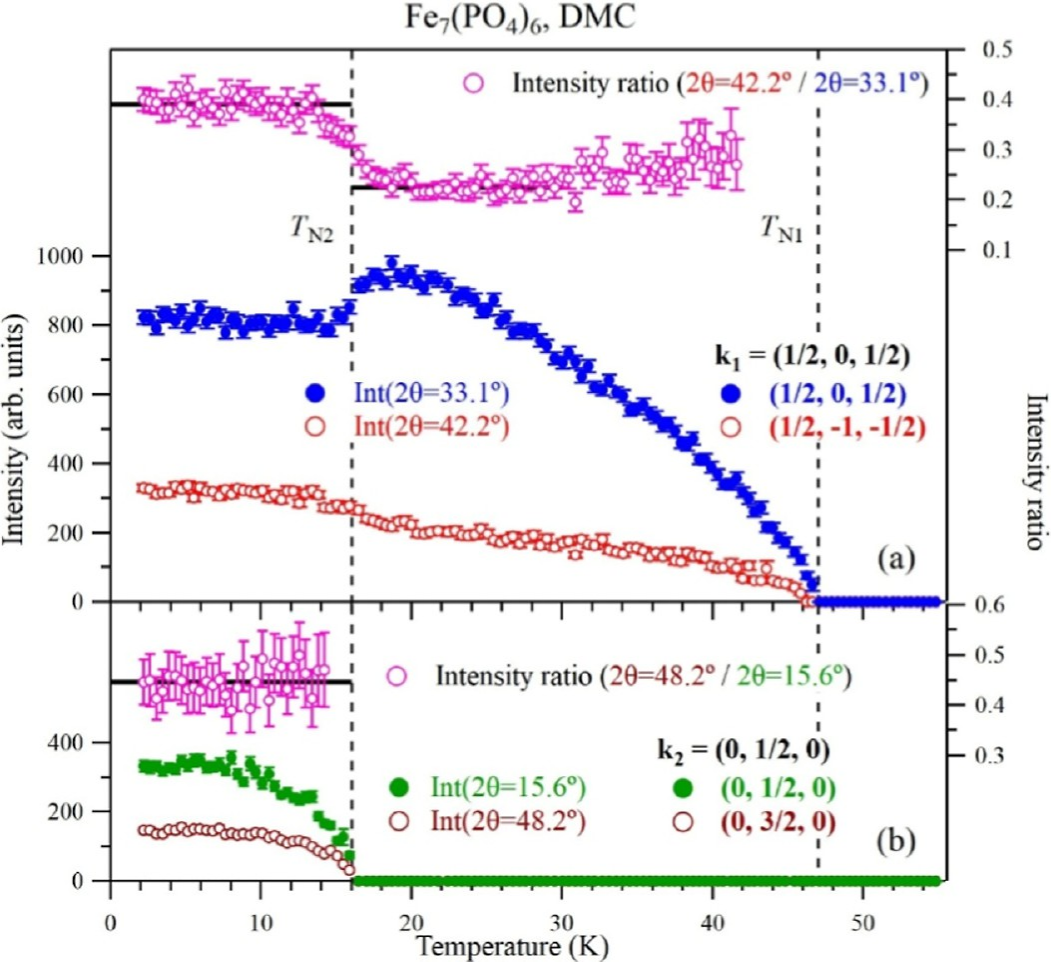
(a,b) Temperature dependence of neutron intensity (left-hand axes) and intensity ratio (right-hand axes) for selected magnetic Bragg peaks of Fe_7_(PO_4_)_6_.

For the triclinic space group *P*1̅ (no. 2), the crystallographic sites 1*a* and 2*i*, and the propagation vectors ***k***_**1**_ and ***k***_**2**_, representation analysis for the possible magnetic structures gives the result summarized in Table 2. Due to the low symmetry of the crystal structure, there are only two irreducible representations (irreps) with different symmetry, which are valid for both propagation vectors ***k***_**1**_ and ***k***_**2**_. For Fe2, Fe3 and Fe4 on site 2*i*, the inversion symmetry from (*x*, *y*, *z*) to (*x̅*, *y̅*, *z̅*) has a ferromagnetic (FM) coupling in *m*U1+ or *m*Y1+, and an AFM coupling in *m*U1– or *m*Y1–. For Fe1 on site 1*a*, magnetic order is possible in *m*U1+ or *m*Y1+, but not in *m*U1– or *m*Y1–. In summary, the refinement of a magnetic structure corresponds to 12 independent fitting parameters (components of magnetic moments) in *m*U1+ or *m*Y1+, and to 9 independent fitting parameters in *m*U1– or *m*Y1–.

**Table 2:** Group Theory Analysis for the Magnetic Structures of Fe_7_(PO_4_)_6_ Phosphate below *T*_N1_ = 47 K and *T*_N2_ = 16 K Calculated Using the Programs ISODISTORT^43,44^ and BASIREPS^42^ ^,^ ^a^

irrep for *k*_1_		(ISODISTORT)	*m*U1+	*m*U1–
irrep for ***k***_**2**_		(ISODISTORT)	*m*Y1+	*m*Y1–
irrep for ***k***_**1**_ and ***k***_**2**_		(BasIreps)	IRrep(1)	IRrep(2)
character set			(1, 1)	(1, −1)
1*a*	Fe1	(0, 0, 0)	(*u*, *v*, *w*)	–
2*i*	Fe2, Fe3, Fe4	(*x*, *y*, *z*)	(*u*, *v*, *w*)	(*u*, *v*, *w*)
		(*x̅*, *y̅*, *z̅*)	(*u*, *v*, *w*)	(−*u*, −*v*, −*w*)

a The triclinic space group is *P*1̅ (no. 2). Magnetic Fe ions are located on sites 1*a* and 2*i*. Magnetic propagation vectors are ***k***_**1**_ = (1/2, 0, 1/2) and ***k***_**2**_ = (0, 1/2, 0). Irrep denotes irreducible representation. Components of the magnetic moments are expressed using (*u*, *v*, *w*). The character sets correspond to the two symmetry elements symm(1): 1 and symm(2): −1 0,0,0.

As shown in Figure 3, the shortest Fe–Fe bond length bond length of 3.11 Å is found in the dimer containing Fe3 at (0.453, 0.114, 0.383) and Fe3′ at (0.547, −0.114, 0.617). Fe3′ is connected to Fe3 by an inversion from (*x*, *y*, *z*) to (*x̅*, *y̅*, *z̅*) and a translation vector of lattice constants (*t*_*x*_, *t*_*y*_, *t*_*z*_) = (1, 0, 1). For both ***k*** vectors, ***k***_**1**_ and ***k***_**2**_, this translation vector gives a FM coupling. Therefore, according to the results of the symmetry analysis (Table 2), the coupling in the Fe3 – Fe3′ dimer is always FM for magnetic structures inside *m*U1+ or *m*Y1+ and always AFM for structures in *m*U1– or *m*Y1–. For the other dimer containing Fe4 at (0.723, 0.529, 0.044) and Fe4′ at (0.277, 0.471, −0.044), the translation vector (1, 1, 0) gives an AFM coupling for both ***k*** vectors, ***k***_**1**_ and ***k***_**2**_. Therefore, the coupling in the Fe4–Fe4′ dimer is always AFM for magnetic structures inside *m*U1+ or *m*Y1+ and always FM for structures in *m*U1– or *m*Y1–. As a result of the symmetry analysis, inside each irrep one dimer has a FM coupling and the other an AFM coupling.

At *T* = 25 K, the AFM structure of Fe_7_(PO_4_)_6_ with propagation vector ***k***_**1**_ = (1/2, 0, 1/2) belongs to the representation *m*U1+. Refinements are shown in Figures 8b and S14b and the results are summarized in Table 3. The strongest magnetic intensity is observed for the Bragg peaks (1/2, 0, 1/2) at 2θ = 33.1°, (1/2, −1, −1/2) at 2θ = 42.2°, and (3/2, −1, −1/2) at 2θ = 56.4° (Figure 8b). All magnetic Fe ions are ordered—with a large moment at the Fe^3+^ sites (4.1 μ_B_ for Fe4 and 3.7 μ_B_ for Fe3), and a much smaller moment at the Fe^2+^ sites (1.7 μ_B_ for Fe1 and 0.7 μ_B_ for Fe2). The noncollinear AFM structure of Fe_7_(PO_4_)_6_ at *T* = 25 K is illustrated in Figure 11. Reflecting the low symmetry of the triclinic crystal structure, directions and magnitudes of the ordered moments all are different for different Fe ions (Fe1, Fe2, Fe3 and Fe4). The AFM structure is dominated by large ordered moments along the *c*-axis (*m*_*z*_ components) with a FM coupling between Fe4 (3.8 μ_B_) and Fe3 (3.3 μ_B_). Within the accuracy of the experimental data, for all Fe ions, rather small components of the ordered moments inside the *ab*-plane exhibit a collinear arrangement at an angle δ ≈ 40° away from the *a*-axis (see dashed line in Figure 11a). This means that all ordered Fe moments lie inside one plane that is defined by the direction of the dashed line in Figure 11a and the *c*-axis.

**Table 3:** Result of the Refinement of the Magnetic Structures of Fe_7_(PO_4_)_6_ Phosphate at *T* = 25 and 2 K Based on Powder Neutron Diffraction Data (DMC, λ = 4.507 Å)^a^ ^b^ ^c^

*T* = 25 K (*T*_N2_ < *T* < *T*_N1_):				
irrep: *m*U1+	*m*_*x*_ (μ_B_)	*m*_*y*_ (μ_B_)	*m*_*z*_ (μ_B_)	*M* (μ_B_)
Fe1 ***k***_1_	1.06(7)	0.63(4)	–0.99(5)	1.72(10)
Fe2 ***k***_1_	–0.41(5)	–0.24(3)	0.45(4)	0.72(7)
Fe3 ***k***_1_	–0.91(5)	–0.54(3)	3.28(4)	3.73(6)
Fe4 ***k***_1_	–0.59(4)	–0.36(3)	3.80(4)	4.07(6)
*R*-factors: *R*_wp_ = 2.25%; *R*_exp_ = 1.24%; *R*_Bragg_ = 1.37% %; *R*_mag(**k**1)_ = 2.08%; χ^2^ = 3.28				

*T* = 2 K (*T* < *T*_N2_):				
irrep: *m*U1+	*m*_*x*_ (μ_B_)	*m*_*y*_ (μ_B_)	*m*_*z*_ (μ_B_)	*M* (μ_B_)
Fe1 ***k***_1_	≈ 0	1.12(7)	–0.28(6)	1.24(10)
Fe2 ***k***_1_	≈ 0	–0.60(5)	0.29(4)	0.75(7)
Fe3 ***k***_1_	≈ 0	–2.11(4)	3.08(5)	4.25(7)
Fe4 ***k***_1_	≈ 0	–1.54(4)	3.61(5)	4.35(7)
irrep: *m*Y1+				
Fe1 ***k***_2_	2.94(5)	–1.45(7)	–2.18(9)	4.28(11)
Fe2 ***k***_2_	–3.99(3)	0.84(5)	0.22(7)	4.32(6)
Fe3 ***k***_2_	–1.31(2)	≈0	≈0	1.31(2)
Fe4 ***k***_2_	–1.04(2)	≈0	≈0	1.04(2)
*R*-factors: *R*_wp_ = 2.80%; *R*_exp_ = 1.25%; *R*_Bragg_ = 1.37% %; *R*_mag(***k*** 1)_ = 2.48%; *R*_mag(***k*** 2)_ = 2.94%; χ^2^ = 4.99				

*T* = 2 K (*T* < *T*_N2_):			
superposition of *k*_1_ and *k*_2_	*M*_max_ (μ_B_)	*M*_min_ (μ_B_)	difference (μ_B_)
Fe1	≈4.7	≈4.2	≈0.5
Fe2	≈4.6	≈4.2	≈0.4
Fe3	≈4.4	≈4.4	≈0.0
Fe4	≈4.6	≈4.4	≈0.2

a Magnetic propagation vectors are ***k***_**1**_ = (1/2, 0, 1/2) and ***k***_**2**_ = (0, 1/2, 0). Components and magnitude of the ordered Fe ions are (*m*_*x*_, *m*_*y*_, *m*_*z*_) *M*.

b Positions of magnetic Fe ions.

c Fe1 (0, 0, 0); Fe2 (0.811, 0.288, 0.281); Fe3 (0.453, 0.114, 0.383); Fe4 (0.723, 0.529, 0.044).

**Figure 11:**
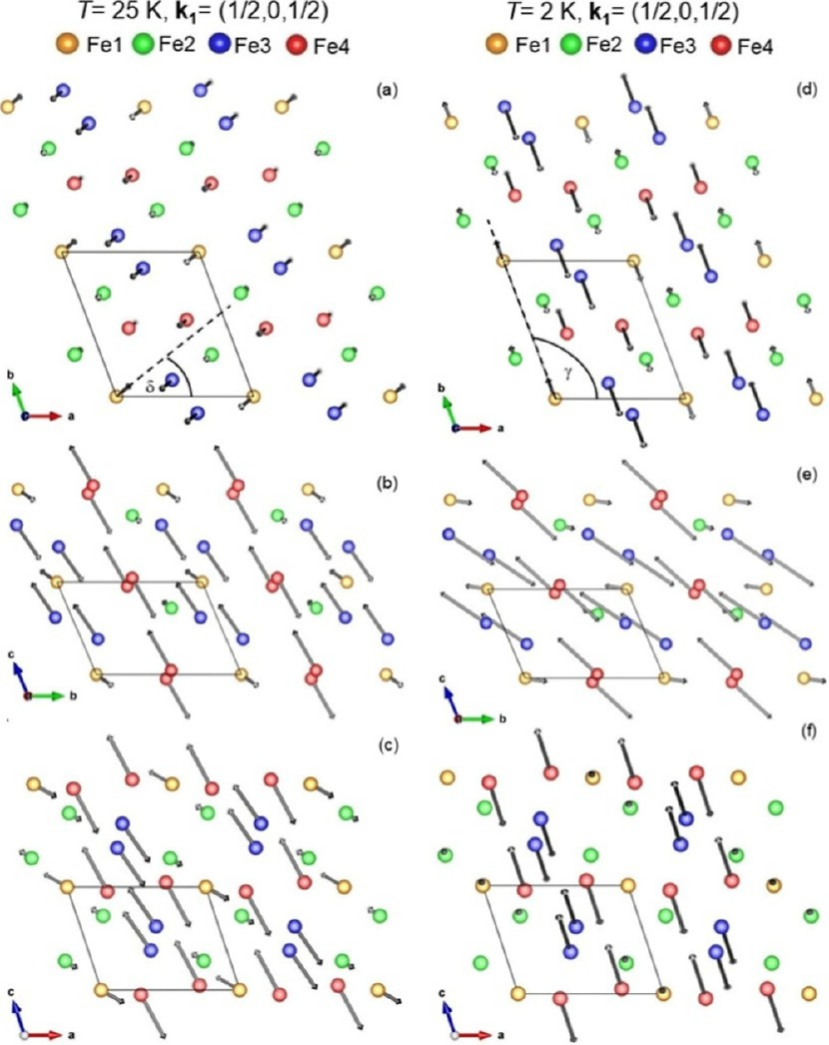
Illustration of the ***k***_**1**_ component of the magnetic structure of Fe_7_(PO_4_)_6_ at *T* = 25 K (a–c) and 2 K (d–f) shown for the components of the ordered magnetic moments inside the *ab*-, *bc*- and *ac*-plane. Drawings were made using VESTA software.^15^

At *T* = 2 K, the AFM structure of Fe_7_(PO_4_)_6_ can be indexed with two propagation vectors ***k***_**1**_ = (1/2, 0, 1/2) and ***k***_**2**_ = (0, 1/2, 0). For ***k***_**1**_ and ***k***_**2**_, the strongest magnetic Bragg peaks appear at different 2θ values. For ***k***_**1**_, they are the same as at *T* = 25 K (Figure 8b). For ***k***_**2**_, the strongest intensity is observed for the magnetic Bragg peaks (0, 1/2, 0) at 2θ = 15.6°, (0, 3/2, 0) at 2θ = 48.2°, and (0, 1/2, 1) at 2θ = 55.1° (Figure 8c). This increases the accuracy of the simultaneous refinement of the two independent magnetic structures for the ***k***_**1**_ and the ***k***_**2**_ components. Refinements are shown in Figures 8c and S14c and the results are summarized in Table 3. The AFM structure belongs to the representation *m*U1+ for ***k***_**1**_ and to *m*Y1+ for ***k***_**2**_. For both components, all magnetic Fe ions are ordered. For the ***k***_**1**_ component at *T* = 2 K, there is a large moment at the Fe^3+^ sites (4.4 μ_B_ for Fe4 and 4.3 μ_B_ for Fe3), and a much smaller moment at the Fe^2+^ sites (1.2 μ_B_ for Fe1 and 0.8 μ_B_ for Fe2). In contrast, for the ***k***_**2**_ component, there is a large moment at the Fe^2+^ sites (4.3 μ_B_ for Fe1 and 4.3 μ_B_ for Fe2), and a much smaller moment at the Fe^3+^ sites (1.0 μ_B_ for Fe4 and 1.3 μ_B_ for Fe3). The magnetic structure of Fe_7_(PO_4_)_6_ at *T* = 2 K is illustrated in Figure 11 for the ***k***_**1**_ component and in Figure 12 for the ***k***_**2**_ component.

**Figure 12:**
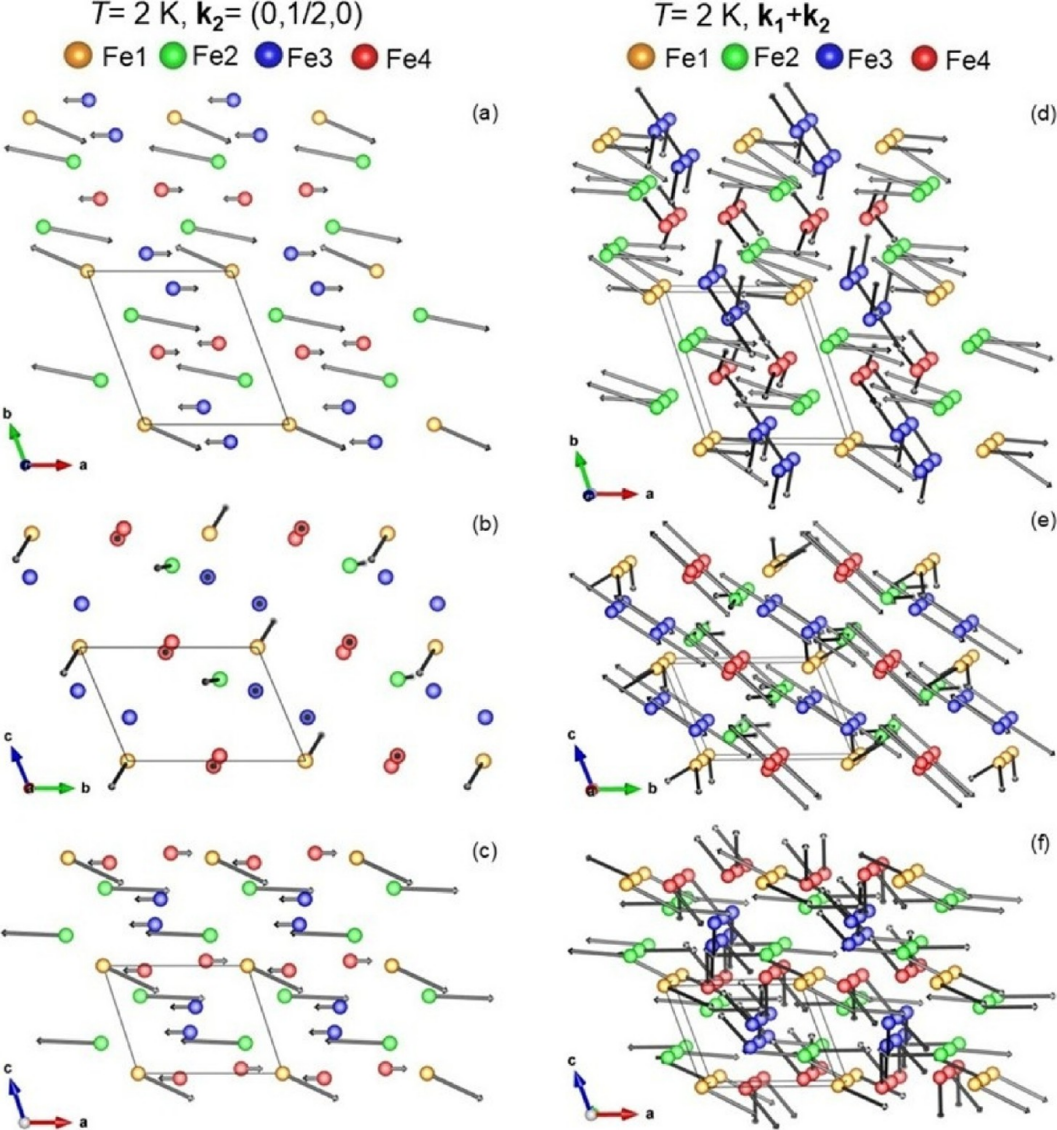
Illustration of the ***k***_**2**_ (a–c) and the ***k***_**1**_ + ***k***_**2**_ (d–f) component of the magnetic structure of Fe_7_(PO_4_)_6_ at *T* = 2 K shown for the components of the ordered magnetic moments inside the *ab*-, *bc*- and *ac*-plane. Drawings were made using VESTA software.^15^

For the ***k***_**1**_ component at *T* = 2 K, within the accuracy of the experimental data, for all Fe ions, the components of the ordered moments inside the *ab*-plane exhibit a collinear arrangement along to the *b*-axis (Figure 11d). This indicates that all ordered moments lie within the *bc*-plane. But directions and magnitudes are all different for different Fe ions (Fe1, Fe2, Fe3 and Fe4). The observed change of intensity of magnetic Bragg peaks near *T*_N2_ = 16 K (Figure 10a) indicates a reorientation of the ordered moments of the ***k***_**1**_ component. A comparison of Figures 11 and 12 suggests that the direction of the collinear arrangement of the components of ordered moments inside the *ab*-plane rotates from an angle δ ≈ 40° at *T* = 25 K to γ = 105° at *T* = 2 K (compare Figure 11a,d). In addition, the large ordered moments at the Fe^3+^ sites, Fe3 and Fe4, rotate away from close to the (0, 0, 1) direction (*c*-axis) at *T* = 25 K toward the (0, −1, 1) direction (the chain direction) at 2 K (compare Figure 11b,e). For the ***k***_**2**_ component at *T* = 2 K, Fe2—located inside the zigzag chain—has a large moment along the *a*-axis (*m*_*x*_ = −4.1 μ_B_)—predominantly perpendicular to the chain direction. Fe1 which connects 4 different chains has large components along the *a*- and *c*-directions (*m*_*x*_ = 2.9 μ_B_, *m*_*z*_ = −2.2 μ_B_).

At *T* = 2 K, the low symmetry of the triclinic crystal structure leads to 24 independent fitting parameters (components of ordered magnetic moments). The quality of the refinement is very good (Figures 8c and S14c) and yields accurate values for the large components of ordered moments (Table 3). However, the many small components are correlated and the available neutron diffraction data do not allow to determine unambiguous values for all 24 moments, because the effective number of measured magnetic Bragg peaks considering instrumental resolution is not large enough. As indicated in Table 3, we have reduced the number of fitting parameters from 24 to 16, by fixing 8 small components to zero (4 for ***k***_**1**_ and 4 for ***k***_**2**_). The result of the 16-parameter refinement is given in Table 3. Both models, with 24 or 16 fitting parameters, give similar agreement values (e.g., χ^2^ values). Figure 12 illustrates the magnetic structure of Fe_7_(PO_4_)_6_ at *T* = 2 K as a superposition of the ***k***_**1**_ and ***k***_**2**_ components. For each magnetic Fe ion, one **k** vector gives rise to a collinear AFM structure with a constant value of the ordered moment. The superposition of ***k***_**1**_ and ***k***_**2**_ usually leads to a noncollinear AFM structure with ordered moments having two different magnitudes *M*_max_ and *M*_min_. The difference between *M*_max_ and *M*_min_ becomes zero if the ordered moments corresponding to ***k***_**1**_ and ***k***_**2**_ are oriented perpendicular to each other. But an angle of exactly 90° is not supported by the symmetry of a triclinic crystal structure with α ≠ 90°, β ≠ 90° and γ ≠ 90°. Estimated values for *M*_max_, *M*_min_ are given in Table 3. They depend on the values of the small components of the ordered moments.

### 3.4 Discussion

The magnetic structure refinements for Fe_7_(PO_4_)_6_ were performed using the ***k***-vector formalism and irreducible representations implemented in FULLPROF.^42^ The corresponding magnetic space groups (MSG) were subsequently assigned, and the magCIF files were generated using ISOCIF from the ISOTROPY Software Suite^43,44^ and MVISUALIZE from the Bilbao Crystallographic Server,^46–48^ following the Guidelines for communicating commensurate magnetic structures.^49^ For the 25 K magnetic phase, the MSG is *P*1̅ (BNS 2.7) and the relation of the magnetic setting to the parent cell is basis = (1, 0, 1), (0, −1, 0), (2, 0, 0), origin = (0.500, 0.000, 0.000). For the 2 K magnetic phase, the MSG is also *P*1̅ (BNS 2.7) and the relation of the magnetic setting to the parent cell is basis = (1, 0, 1), (1, 0, −1), (2, 2, 0), origin = (0.500, 0.500, 0.000). The magCIF files generated by the software MVISUALIZE from the Bilbao Crystallographic Server are printed in Tables S7 (for *T* = 25 K) and S8 (for *T* = 2 K).

Magnetic ordering in the MV compound Fe_7_(PO_4_)_6_ occurs in two successive AFM phase transitions at *T*_N1_ = 47 K and *T*_N2_ = 16 K. Large ordered moments appear first at the two Fe^3+^ sites (below *T*_N1_ with a propagation vector ***k***_**1**_ = (1/2, 0, 1/2)) and then at the two Fe^2+^ sites (below *T*_N2_ with a different propagation vector ***k***_**2**_ = (0, 1/2, 0)). Besides the large ordered moments, much smaller moments are induced at the Fe^2+^ sites (below *T*_N1_ with ***k***_**1**_) and at the Fe^3+^ sites (below *T*_N2_ with ***k***_**2**_). At low temperature, this results in a complex magnetic structure with nonconstant total ordered moments at each site. A somewhat similar situation was observed, for example, in mineral ilvaite, Ca­(Fe^2+^,Fe^3+^)­Fe^3+^Si_2_O_7_O­(OH),^21^ where the first magnetic transition takes place at 116 K, but one site with Fe^2+^ remains completely disordered (even without any detectable induced moments), and it is ordered only below the second magnetic transition at 40 K.

The Fe^3+^ cations reach nearly maximum saturation values quite fast, just above *T*_N2_. On the other hand, the Fe^2+^ cations are ordered with significantly reduced moments just above *T*_N2_ probably because they prefer different orientations with the propagation vector ***k***_**2**_. This fact can explain why magnetic susceptibilities do not drop at *T*_N1_ as one could expect for an AFM transition, but continue the paramagnetic trend with decreasing temperature.^18,19^ Magnetic susceptibilities only drop below *T*_N2_ (Figure 6).

The magnetic structure of Fe_7_(PO_4_)_6_ belongs to the irreps *m*U1+ (for ***k***_**1**_) and *m*Y1+ (for ***k***_**2**_), where the symmetry requests an AFM coupling in the Fe4–Fe4′ dimer and a FM coupling in the Fe3–Fe3′ dimer. According to the Goodenough–Kanamori rules, the AFM exchange is always expected for Fe^3+^–Fe^3+^ bonds independent of Fe–O–Fe angles.^50^ However, the strength of AFM interactions strongly depends on Fe–O–Fe angles, where the strongest AFM interactions are expected for the 180° bonds. Our magnetic structures of Fe_7_(PO_4_)_6_ revealed that the AFM interactions are realized in the Fe4–Fe4′ dimer unit with the larger bond angle of 105.1(3)^°^ (Table S1). On the other hand, the Fe3–Fe3′ dimer unit with the smaller bond angle of 98.1(3)^°^ (Table S1) has the FM exchange forced by the overall magnetic structure because it is easier to force FM exchange in the Fe3–Fe3′ dimer.

The magnetic structures found in our work are partly consistent with the previous Mössbauer spectroscopy results.^19^ It was found that just above *T*_N2_, the hyperfine field values on Fe^3+^ cations reached almost saturation values corresponding to nearly full ordered moments. On the other hand, the hyperfine field values on Fe^2+^ cations were significantly reduced in agreement with the significantly reduced moments found in our work. Below *T*_N2_, the hyperfine field value on one Fe^2+^ site increased rapidly in agreement with the large ordered moment. On the other hand, the hyperfine field value on the second Fe^2+^ site remained small,^19^ while our results show the large ordered moments for both Fe^2+^ sites. This fact could call for the reanalysis of the previous Mössbauer spectroscopy results. However, we note that for Fe^2+^ cations there are not so strong correlations between ordered moments and hyperfine fields in comparison with Fe^3+^ cations.

The ordered moments on Fe^2+^ cations in Fe_7_(PO_4_)_6_ are found to be slightly higher than the spin-only values of 4 μ_B_. This fact shows that there are noticeable contributions from spin–orbital coupling. Such a coupling can increase ordered moments on Fe^2+^ cations up to 4.5 μ_B_.^21^ Examples of increased Fe^2+^ ordered moments have been reported for K_4_Fe_3_F_12_ (∼4.3 μ_B_)^51^ and FeF_2_ (∼4.5 μ_B_).^52,53^

## 4 Conclusion

The triclinic crystal-structure type of the MV (Fe^2+^, Fe^3+^) compound Fe_7_(PO_4_)_6_ is adapted by other phosphates, vanadates, molybdates, and arsenates. Some materials with this crystal-structure type have attracted interest for applications in batteries and Na-ion intercalation, for use as colorful pigments and because of multiferroic properties. For the parent compound Fe_7_(PO_4_)_6_, we have determined complex magnetic structures by powder neutron diffraction and constructed a temperature-magnetic field phase diagram based on temperature- and field-dependent magnetization and specific heat measurements. At zero field, Fe_7_(PO_4_)_6_ shows two successive AFM phase transitions at *T*_N1_ and *T*_N2_ with different propagation vectors ***k***_**1**_ and ***k***_**2**_. Below *T*_N1_, Fe^3+^ cations order with ***k***_**1**_ and adopt large moments with inducing small moments at the Fe^2+^ cations. Below *T*_N2_, Fe^2+^ cations order with ***k***_**2**_ and adopt large moments with inducing small moments at the Fe^3+^ cations. The ***k***_**1**_ component shows a reorientation near *T*_N2_ and coexists with the ***k***_**2**_ component at lower temperatures. Our results showed the complexity of the magnetic structures and magnetic phase diagram of the MV phosphate Fe_7_(PO_4_)_6_. It may be interesting to track changes in magnetic structures and magnetic phase diagrams with changes in the Fe^3+^:Fe^2+^ ratio in Fe_7_H_*x*_(PO_4_)_6_ or other doped variants.

## Supplementary Material







## Notes

Details of *C*_p_ vs *T*, *M* vs *H* curves, *M* vs *T* curves, and differential curves, refinement of neutron diffraction patterns, Tables with interatomic distances and bond angles, Tables with numerical values used to plot Figure 7, printouts of two magCIF files describing the magnetic structures at 25 and 2 K using magnetic space groups (PDF)
Data for the magnetic structure at *T* = 25 K (MCIF)
Data for the magnetic structure at *T* = 2 K (MCIF)
