# Supplementary material for: Magnetic Structures and Magnetic Phase Diagram of the Mixed-Valence Iron Phosphate Fe7(PO4)6
Source: Inorg Chem. 2026 Mar 26;65(13):7418–30. doi: 10.1021/acs.inorgchem.6c00534 (PMC13058894; doi:10.1021/acs.inorgchem.6c00534)
Supplement: Supplementary file 1 [file ic6c00534_si_001.pdf]

Supporting Information (Online Material) for

Magnetic Structures and Magnetic Phase Diagram of the Mixed-Valence Iron Phosphate $Fe_7(PO_4)_6$

Andreas Dönni,[1] Lukas Keller,[2] Vladimir Y. Pomjakushin,[2] Naohito Tsujii,[1]
and Alexei A. Belik[1,*]

[1] *Research Center for Materials Nanoarchitectonics (MANA), National Institute for Materials Science (NIMS), Namiki 1-1, Tsukuba, Ibaraki 305-0044, Japan*

[2] *PSI Center for Neutron and Muon Sciences, CH-5232 Villigen PSI, Switzerland*

* Corresponding Author: Alexei.Belik@nims.go.jp

Table S1: Fe–Fe bond lengths (up to a distance of 3.6 Å) and corresponding Fe–O–Fe bond angles of paramagnetic $Fe_7(PO_4)_6$ phosphate at T = 60 K based on powder neutron diffraction data (HRPT, λ = 1.886 Å).

Fe – Fe bond length (Å)		Fe–O–Fe bond angles (deg)	
Fe3 – Fe3 (1x)	3.110(4) Å	Fe3-O2-Fe3 (2x)	98.1(3) °
Fe2 – Fe3 (1x)	3.185(4) Å	Fe2-O5-Fe3 (1x)	100.9(2) °
		Fe2-O8-Fe3 (1x)	101.8(3) °
Fe2 – Fe4 (1x)	3.215(4) Å	Fe2-O9-Fe4 (1x)	102.6(3) °
		Fe2-O10-Fe4 (1x)	99.9(3) °
Fe4 – Fe4 (1x)	3.343(4) Å	Fe4-O6-Fe4 (2x)	105.1(3) °
Fe1 – Fe2 (2x)	3.580(3) Å	Fe1-O1-Fe2 (1x)	114.6(2) °
Fe1 – Fe3 (2x)	3.588(3) Å	Fe1-O11-Fe3 (1x)	114.6(2) °

Table S2: Fe–O bond lengths up to 2.5 Å of paramagnetic $Fe_7(PO_4)_6$ phosphate at T = 60 K based on powder neutron diffraction data (HRPT, λ = 1.886 Å).

Fe – O bond length (Å)			Fe – O bond length (Å)		
Fe1	– O7 (2x)	2.042(4) Å	Fe3	– O4 (1x)	1.863(5) Å
	– O11 (2x)	2.237(4) Å		– O11 (1x)	2.024(4) Å
	– O1 (2x)	2.265(3) Å		– O8 (1x)	2.028(5) Å
Fe2	– O1 (1x)	1.986(4) Å		– O2 (1x)	2.040(5) Å
	– O8 (1x)	2.077(4) Å		– O5 (1x)	2.048(4) Å
	– O5 (1x)	2.081(5) Å		– O2 (1x)	2.078(5) Å
	– O10 (1x)	2.110(5) Å	Fe4	– O3 (1x)	1.904(5) Å
	– O9 (1x)	2.124(4) Å		– O12 (1x)	1.929(3) Å
Average Fe1 – O		2.181 Å (of 6)		– O9 (1x)	1.995(4) Å
Average Fe2 – O		2.076 Å (of 5)		– O6 (1x)	2.017(5) Å
Average Fe3 – O		2.014 Å (of 6)		– O10 (1x)	2.089(5) Å
Average Fe4 – O		2.021 Å (of 6)		– O6 (1x)	2.191(4) Å

Table S3: P–O bond lengths (Å) and corresponding O–P–O bond angles (deg) in tetrahedra of paramagnetic $Fe_7(PO_4)_6$ phosphate at $T = 60$ K based on powder neutron diffraction data (HRPT, λ = 1.886 Å).

P – O bond length (Å)			O–P–O bond angles (deg)	
P1	– O7 (1x)	1.506(5) Å	O2 – P1 – O6 (1x)	106.5(4) °
	– O6 (1x)	1.552(6) Å	O2 – P1 – O7 (1x)	111.7(4) °
	– O2 (1x)	1.555(5) Å	O2 – P1 – O8 (1x)	107.4(4) °
	– O8 (1x)	1.557(5) Å	O6 – P1 – O7 (1x)	114.6(4) °
			O6 – P1 – O8 (1x)	108.6(5) °
			O7 – P1 – O8 (1x)	107.8(4) °
P2	– O3 (1x)	1.504(6) Å	O1 – P1 – O3 (1x)	111.2(4) °
	– O4 (1x)	1.530(6) Å	O1 – P1 – O4 (1x)	107.8(4) °
	– O1 (1x)	1.536(4) Å	O1 – P1 – O10 (1x)	110.8(3) °
	– O10 (1x)	1.544(5) Å	O3 – P1 – O4 (1x)	111.9(5) °
			O3 – P1 – O10 (1x)	107.1(4) °
			O4 – P1 – O10 (1x)	108.1(5) °
P3	– O12 (1x)	1.499(5) Å	O5 – P3 – O9 (1x)	109.5(4) °
	– O9 (1x)	1.540(5) Å	O5 – P3 – O11 (1x)	109.6(5) °
	– O5 (1x)	1.546(5) Å	O5 – P3 – O12 (1x)	109.9(4) °
	– O11 (1x)	1.561(6) Å	O9 – P3 – O11 (1x)	107.7(4) °
			O9 – P3 – O12 (1x)	110.9(4) °
			O11 – P3 – O12 (1x)	109.3(4) °

Table S4: Magnetic anomalies in $Fe_7(PO_4)_6$ from specific heat measurements.

H (kOe)	T_{N2} (K)	T_{N2b} (K)	Note for T_{N2b}	T_{N1} (K)
0	16.4			47
10	16.2			
20	16.2			
30	16.1	15.0		
35	16.1	14.5		
40	16.1	14.0		
45	16.0	13.3	very weak	
50	16.0			
55	15.9			
60	15.9			
65	15.9	15.6		
70	16.0	15.6		47
75	15.9	15.4		
80	15.9	15.1		
85	15.9	14.9		
90	15.8	14.4		47

Table S5: Magnetic anomalies in $Fe_7(PO_4)_6$ from M versus H curves.

T (K)	H (kOe)
1.7	55.5
1.8	55.5
2	55.5
3	55.5
4	55.5
5	56.0
6	56.5
7	56.5
8	56.5
9	56.5
10	55.5
11	54.0
12	51.0
13	47.0
14	41.5
14.5	37.5
15	33.0
15.5	26.5
16	17.0
17	no anomalies

Table S6: Magnetic anomalies in $Fe_7(PO_4)_6$ from χ versus T curves.

H (kOe)	T_{N2b} (K)	T_{ZFC} (K)	T_{N1} (K)
70.0		8.0	47
67.5		8.0	
65.0		8.0	
62.5		8.0	
60.0		8.0	47
57.5		8.0	
55.0	10.0	8.2	
52.5	11.4	8.2	
50.0	12.2	8.2	47
47.5	12.8	8.2	
45.0	13.2	8.2	
42.5	13.8	8.2	
40.0	14.2		47
37.5	14.4		
35.0	14.7		
32.5	15.0		
30.0	15.2		47
25.0			
20.0			47
10.0			47
1.0			47

Table S7: Printout of the magCIF file (all ISO) for the magnetic structure of $Fe_7(PO_4)_6$ at T= 25 K generated by the software MVISUALIZE from the Bilbao Crystallographic Server.

```
#\#CIF_2.0
# Created by the Bilbao Crystallographic Server
# http://www.cryst.ehu.es
# Date: 03/13/2026 14:58:19

data_Fe7_PO4_6_magnetic_25K
_audit_creation_date            2026-03-13
_audit_creation_method          "Bilbao Crystallographic Server"

_chemical_name_systematic
;
;
_chemical_name_common                  "Fe7(PO4)6"
_chemical_formula_moiety               ?
_chemical_formula_structural           ?
_chemical_formula_analytical           ?
_chemical_formula_iupac                ?
_chemical_formula_sum                  "Fe7 P6 O24"
_chemical_formula_weight               ?
_chemical_melting_point                ?
_chemical_compound_source              ?
_chemical_absolute_configuration       ?


_citation_journal_abbrev        "Inorg. Chem."
_citation_journal_volume        ?
_citation_page_first            ?
_citation_page_last             ?
_citation_article_id            ?
_citation_year                  2026
_citation_DOI                   ?

loop_
_citation_author_name
"A. Dönni"
"L. Keller"
"V. Yu. Pomjakushin"
"N. Tsujii"
"A. A. Belik"

_temperature_position_structure        25
_temperature_atom_positions            60
_temperature_non-magn_atom_positions   60
_temperature_cell_parameters           60

_atomic_positions_source_database_code_ICSD  ?
_atomic_positions_source_other               "Rietveld refinement of neutron powder diffraction data"
```

_transition_temperature 47
_experiment_temperature 25

loop_
_irrep_id
_irrep_dimension
_irrep_small_dimension
_irrep_direction_type
_irrep_action
_irrep_modes_number
_irrep_presence
mU1+ 1 1 special primary 12 .

_exptl_crystal_magnetic_properties_details
;
Neutron powder diffraction (DMC, PSI)
;

_active_magnetic_irreps_details
;
At 25 K, the magnetic structure is described by the propagation vector k1 = (1/2, 0, 1/2) and irrep mU1+.
;

_parent_space_group.name_H-M_alt 'P -1'
_parent_space_group.IT_number 2
_parent_space_group.transform_Pp_abc 'a,b,c;0,0,0'

loop_
_parent_propagation_vector.id
_parent_propagation_vector.kxkykz
k1 [1/2 0 1/2]

_parent_space_group.child_transform_Pp_abc ?
_space_group_magn.transform_BNS_Pp_abc ?

_space_group_magn.number_BNS 2.7
_space_group_magn.name_BNS "P_S -1"
_space_group_magn.point_group_name "-11'"
_space_group_magn.point_group_number "2.2.4"
_cell_length_a 9.13229
_cell_length_b 9.30538
_cell_length_c 15.92966
_cell_angle_alpha 74.77760
_cell_angle_beta 42.95663
_cell_angle_gamma 63.40064

loop_
_space_group_symop_magn_operation.id
_space_group_symop_magn_operation.xyz
1 x,y,z,+1
2 -x,-y,-z,+1

loop_

```
_space_group_symop_magn_centering.id
_space_group_symop_magn_centering.xyz
1 x,y,z,+1
2 x,y,z+1/2,-1

loop_
_atom_site_label
_atom_site_type_symbol
_atom_site_fract_x
_atom_site_fract_y
_atom_site_fract_z
_atom_site_occupancy
Fe1 Fe 0.00000 0.00000 0.00000 1
Fe2 Fe 0.28100 0.71245 0.76489 1
Fe3 Fe 0.38282 0.88591 0.53496 1
Fe4 Fe 0.04426 0.47094 0.83918 1
P1 P 0.09646 0.16694 0.74865 1
P2 P 0.39880 0.62942 0.41649 1
P3 P 0.22848 0.23329 0.46126 1
O1 O 0.27530 0.75894 0.38224 1
O2 O 0.31200 0.08305 0.61364 1
O3 O 0.25398 0.53491 0.51478 1
O4 O 0.44944 0.71448 0.45835 1
O5 O 0.45746 0.22723 0.40673 1
O6 O 0.06650 0.34615 0.74179 1
O7 O 0.11990 0.07972 0.83457 1
O8 O 0.12016 0.83912 0.70598 1
O9 O -0.01920 0.65754 0.41485 1
O10 O 0.36640 0.49267 0.69720 1
O11 O 0.22574 0.06031 0.48805 1
O12 O 0.21102 0.29457 0.87088 1

loop_
_atom_site_moment.label
_atom_site_moment.crystalaxis_x
_atom_site_moment.crystalaxis_y
_atom_site_moment.crystalaxis_z
_atom_site_moment.symmform
_atom_site_moment.magnitude
_atom_site_moment.spherical_azimuthal
_atom_site_moment.spherical_polar
Fe1 1.42152 0.633 -2.29479 Mx,My,Mz ? ? ?
Fe2 0.64536 0.244 -0.96886 Mx,My,Mz ? ? ?
Fe3 4.71587 0.544 -5.02 Mx,My,Mz ? ? ?
Fe4 5.45609 0.356 -5.35259 Mx,My,Mz ? ? ?
```

Table S8: Printout of the magCIF file (all ISO) for the magnetic structure of $Fe_7(PO_4)_6$ at T= 2 K generated by the software MVISUALIZE from the Bilbao Crystallographic Server.

```
#\#CIF_2.0
# Created by the Bilbao Crystallographic Server
# http://www.cryst.ehu.es
# Date: 03/13/2026 14:37:14

data_Fe7_PO4_6_2K_all_ISO_mvis_cleaned
_audit_creation_date          2026-03-13
_audit_creation_method         "Bilbao Crystallographic Server"

_chemical_name_systematic
;
Mixed-valence iron phosphate
;
_chemical_name_common                  "Fe7(PO4)6"
_chemical_formula_moiety               ?
_chemical_formula_structural           "Fe7(PO4)6"
_chemical_formula_analytical           ?
_chemical_formula_iupac                ?
_chemical_formula_sum                  "Fe7 P6 O24"
_chemical_formula_weight               ?
_chemical_melting_point                ?
_chemical_compound_source              "Solid-state synthesis from FePO4 and Fe"
_chemical_absolute_configuration       ?


_citation_journal_abbrev        "Inorg. Chem."
_citation_journal_volume        ?
_citation_page_first            ?
_citation_page_last             ?
_citation_article_id            ?
_citation_year                  2026
_citation_DOI                   ?

loop_
_citation_author_name
"Andreas Dönni"
"Lukas Keller"
"V. Yu. Pomjakushin"
"Naohito Tsujii"
"Alexei A. Belik"

_temperature_position_structure         60
_temperature_atom_positions             60
_temperature_non-magn_atom_positions    60
_temperature_cell_parameters            60

_atomic_positions_source_database_code_ICSD  ?
```

_atomic_positions_source_other "Rietveld refinement of HRPT neutron powder diffraction data at 60 K; positions fixed in DMC magnetic refinement at 2 K"

_transition_temperature 16
_experiment_temperature 2

loop_
_irrep_id
_irrep_dimension
_irrep_small_dimension
_irrep_direction_type
_irrep_action
_irrep_modes_number
_irrep_presence
mU1+ 1 1 special primary ? .
mY1+ 1 1 special secondary ? .

_exptl_crystal_magnetic_properties_details
;
Powder neutron diffraction (DMC, PSI), lambda = 4.507 A. The magnetic structure at 2 K is represented as an explicit real-space superposition of the k1 and k2 components in a commensurate magnetic supercell.
;

_active_magnetic_irreps_details
;
At 2 K the magnetic structure is described by the coexistence of two propagation vectors, k1 = (1/2, 0, 1/2) and k2 = (0, 1/2, 0). The corresponding irreducible representations used in the refinement are mU1+ for k1 and mY1+ for k2. The moment values listed below correspond to the explicit superposition of the two components in the final magnetic cell.
;

_parent_space_group.name_H-M_alt 'P -1'
_parent_space_group.IT_number 2
_parent_space_group.transform_Pp_abc 'a,b,c;0,0,0'

loop_
_parent_propagation_vector.id
_parent_propagation_vector.kxkykz
k1 [1/2 0 1/2]
k2 [0 1/2 0]

_parent_space_group.child_transform_Pp_abc ?
_space_group_magn.transform_BNS_Pp_abc ?

_space_group_magn.number_BNS 2.7
_space_group_magn.name_BNS "P_S -1"
_space_group_magn.point_group_name "-11'"
_space_group_magn.point_group_number "2.2.4"
_cell_length_a 9.14305
_cell_length_b 11.15869
_cell_length_c 21.10500
_cell_angle_alpha 51.72639
_cell_angle_beta 80.92189

_cell_angle_gamma 76.89868

loop_
_space_group_symop_magn_operation.id
_space_group_symop_magn_operation.xyz
1 x,y,z,+1
2 -x,-y,-z,+1

loop_
_space_group_symop_magn_centering.id
_space_group_symop_magn_centering.xyz
1 x,y,z,+1
2 x,y,z+1/2,-1

loop_
_atom_site_label
_atom_site_type_symbol
_atom_site_fract_x
_atom_site_fract_y
_atom_site_fract_z
_atom_site_occupancy
Fe1 Fe 0.00000 0.00000 0.00000 1
Fe2 Fe 0.50000 0.50000 0.00000 1
Fe3 Fe 0.40212 0.12112 0.64377 1
Fe4 Fe -0.09788 0.62112 0.64377 1
Fe5 Fe 0.36074 -0.02208 0.55704 1
Fe6 Fe 0.86074 0.47792 0.55704 1
Fe7 Fe 0.11891 0.07465 0.76453 1
Fe8 Fe 0.61891 0.57465 0.76453 1
P1 P -0.07142 0.83212 -0.08347 1
P2 P 0.42858 0.33212 -0.08347 1
P3 P 0.13000 0.73120 0.68529 1
P4 P 0.63000 0.23120 0.68529 1
P5 P 0.80638 0.57790 0.88336 1
P6 P 0.30638 0.07790 0.88336 1
O1 O 0.03701 0.76171 0.62053 1
O2 O 0.53701 0.26171 0.62053 1
O3 O -0.03284 0.65516 -0.04152 1
O4 O 0.46716 0.15516 -0.04152 1
O5 O 0.03622 0.78224 0.73254 1
O6 O 0.53622 0.28224 0.73254 1
O7 O 0.26503 0.81559 0.64276 1
O8 O 0.76503 0.31559 0.64276 1
O9 O -0.02219 0.52035 0.88638 1
O10 O 0.47781 0.02035 0.88638 1
O11 O -0.01864 -0.08514 0.82693 1
O12 O 0.48136 0.41486 0.82693 1
O13 O -0.00567 0.87443 -0.03986 1
O14 O 0.49433 0.37443 -0.03986 1
O15 O 0.24570 0.12554 0.58044 1
O16 O 0.74570 0.62554 0.58044 1
O17 O 0.72442 0.74362 0.67123 1
O18 O 0.22442 0.24362 0.67123 1
O19 O 0.30994 -0.05646 0.75366 1

O20 O 0.80994 0.44354 0.75366 1
O21 O 0.74395 0.51821 -0.03016 1
O22 O 0.24395 0.01821 -0.03016 1
O23 O 0.22919 0.01817 0.85271 1
O24 O 0.72919 0.51817 0.85271 1

loop_
_atom_site_moment.label
_atom_site_moment.crystalaxis_x
_atom_site_moment.crystalaxis_y
_atom_site_moment.crystalaxis_z
_atom_site_moment.symmform
_atom_site_moment.magnitude
_atom_site_moment.spherical_azimuthal
_atom_site_moment.spherical_polar
Fe1 -0.0758 -4.41309 0.37166 Mx,My,Mz ? ? ?
Fe2 -1.57935 -5.26571 2.91329 Mx,My,Mz ? ? ?
Fe3 -2.03623 -3.37889 0.26948 Mx,My,Mz ? ? ?
Fe4 -3.04803 -3.58404 1.63126 Mx,My,Mz ? ? ?
Fe5 2.50027 -2.35284 -2.39155 Mx,My,Mz ? ? ?
Fe6 -3.99996 0.52254 2.39155 Mx,My,Mz ? ? ?
Fe7 2.75394 -2.97273 -1.74479 Mx,My,Mz ? ? ?
Fe8 -3.9475 1.51605 1.74479 Mx,My,Mz ? ? ?

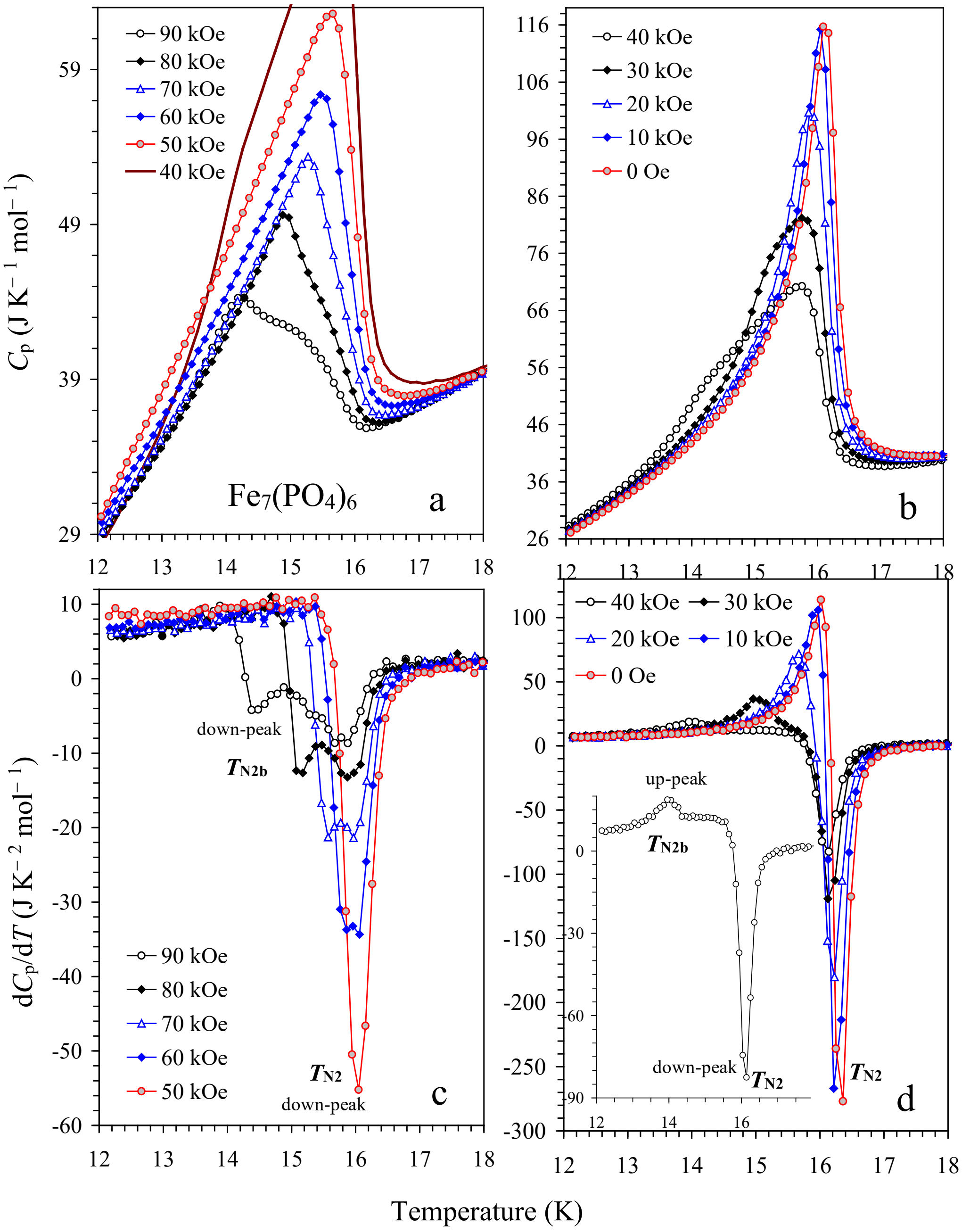

Figure S1. (a) and (b) Specific heat, C_p versus T, at different magnetic fields for $Fe_7(PO_4)_6$, measured on cooling. (c) and (d) Differential dC_p/dT versus T curves at the same fields. Inset in (d) shows dC_p/dT versus T curve at H = 40 kOe.

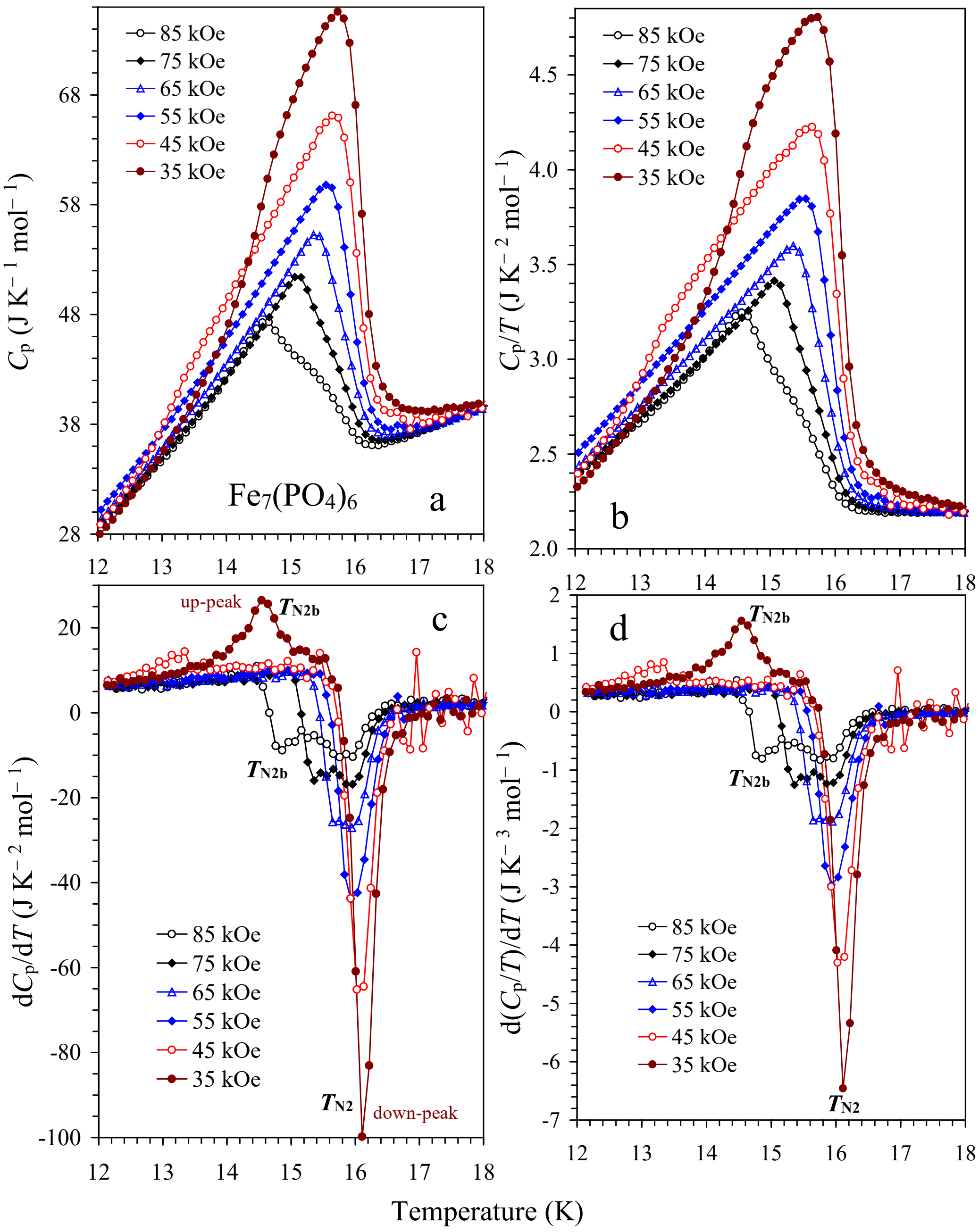

Figure S2. (a) Specific heat, C_p versus T, at different magnetic fields for $Fe_7(PO_4)_6$, measured on cooling. (b) The same curves plotted as C_p/T versus T. (c) The same curves plotted as dC_p/dT versus T. (d) The same curves plotted as $d(C_p/T)dT$ versus T.

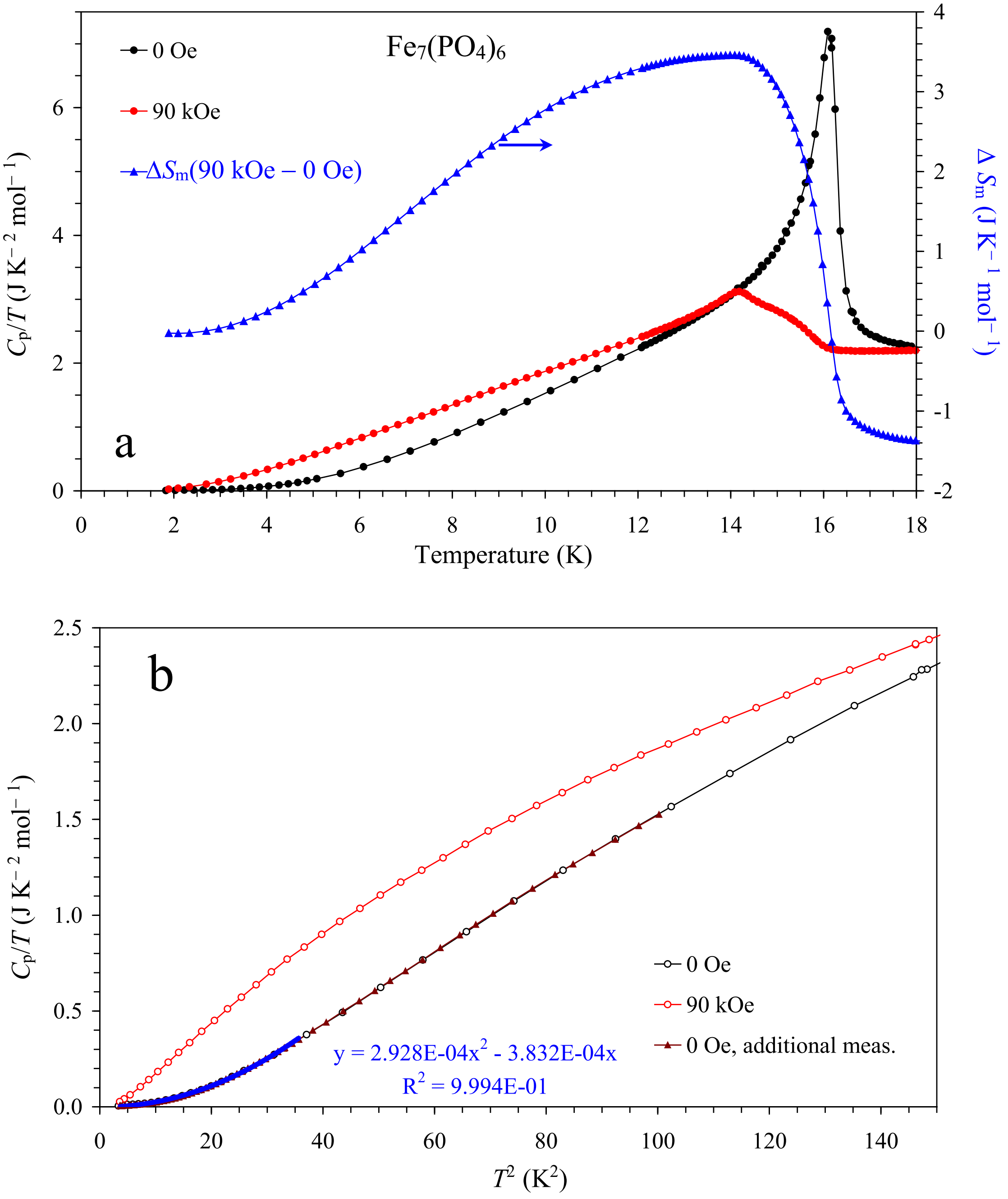

Figure S3. (a) Specific heat, C_p/T versus T, at $H = 0$ Oe and 90 kOe (the left-hand axis) and the entropy change (ΔS_m(90 kOe – 0 Oe) (the right-hand axis) for $Fe_7(PO_4)_6$.
(b) C_p/T versus T^2 plots at $H = 0$ Oe and 90 kOe; this plot shows that there is almost no linear behavior at $H = 0$ Oe; on the other hand, this plot shows that the electronic contribution at $H = 0$ Oe is negligible in agreement with insulating properties.

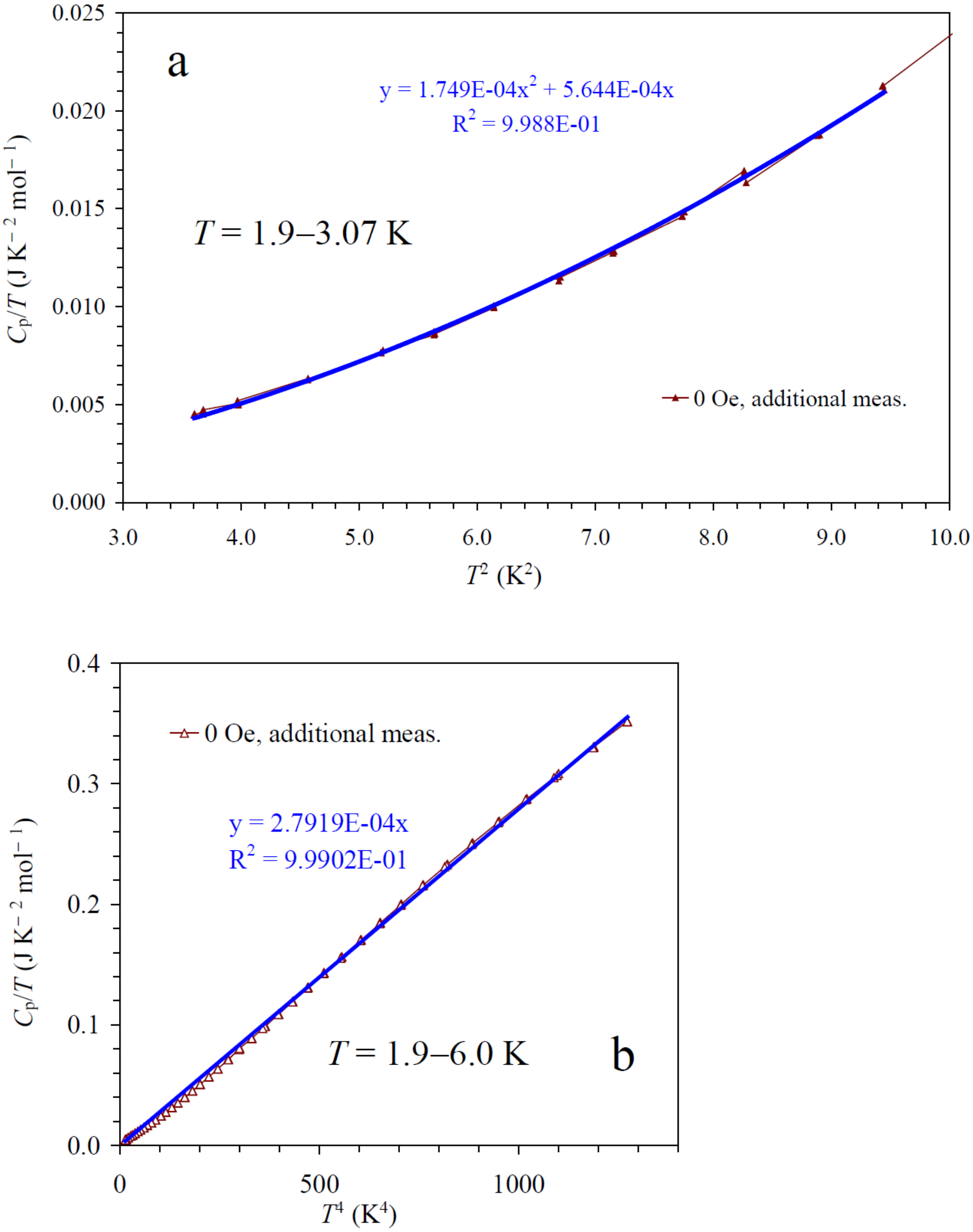

Figure S4. (a) A C_p/T versus T^2 plot at $H = 0$ Oe with the fit (between 1.9 K and 3.07 K; blue line) and fit equation.

(b) A C_p/T versus T^4 plot at $H = 0$ Oe with the fit (between 1.9 K and 6.0 K; blue line) and fit equation.

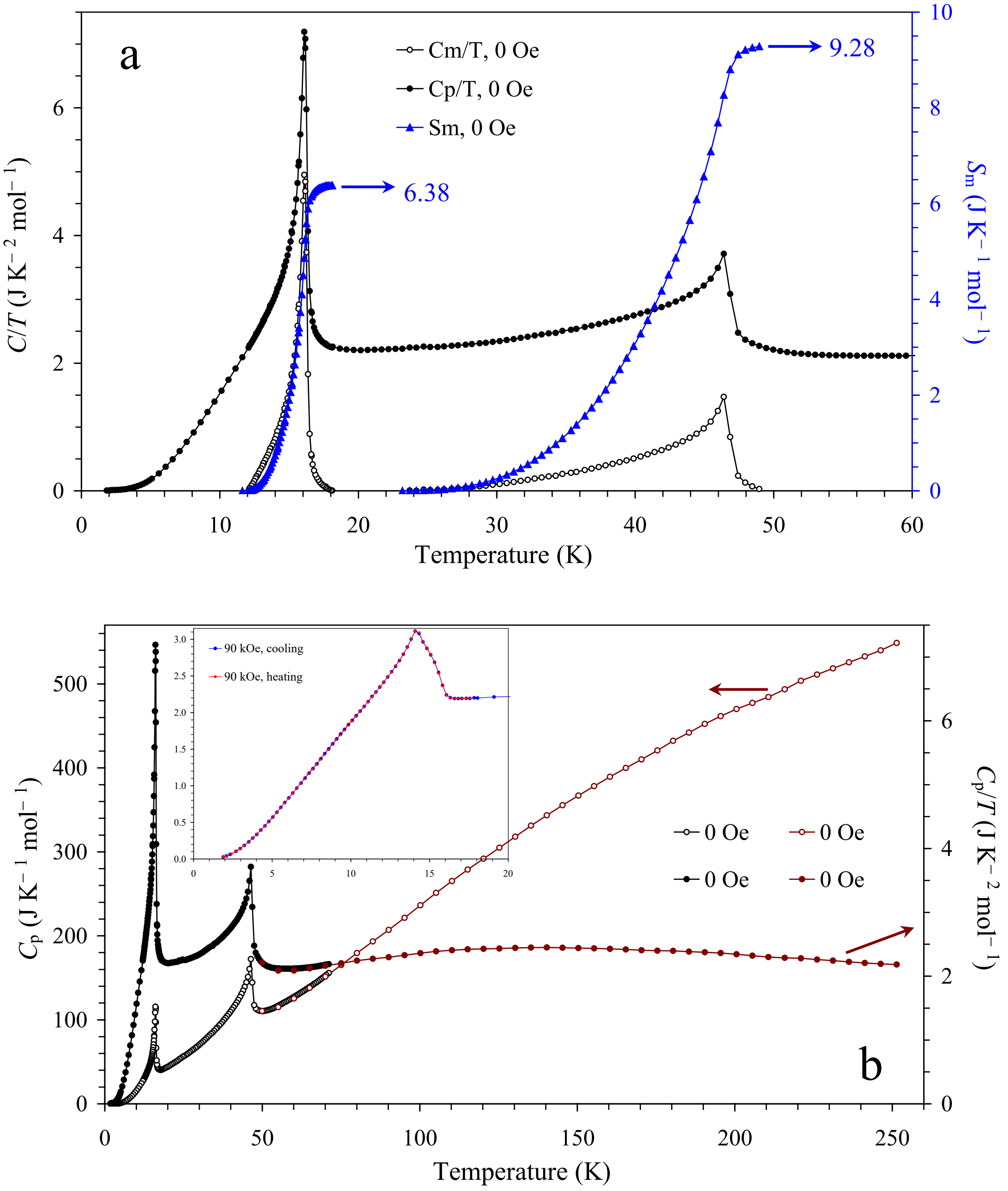

Figure S5. (a) Specific heat, C_p/T versus T and C_m/T versus T, at H = 0 Oe (the left-hand axis) and the magnetic entropy, S_m versus T (the blue curve), (the right-hand axis) for $Fe_7(PO_4)_6$. C_m is "magnetic" specific heat obtained assuming the "lattice" contribution ($C_{lattice}/T$ = 2.24 JK^{-2}mol^{-1}) near the peaks.

(b) C_p versus T (the left-hand axis) and C_p/T versus T (the right-hand axis) curves at H = 0 Oe up to 250 K. The inset shows C_p/T versus T curves at H = 90 kOe on cooling and heating (there was no hysteresis).

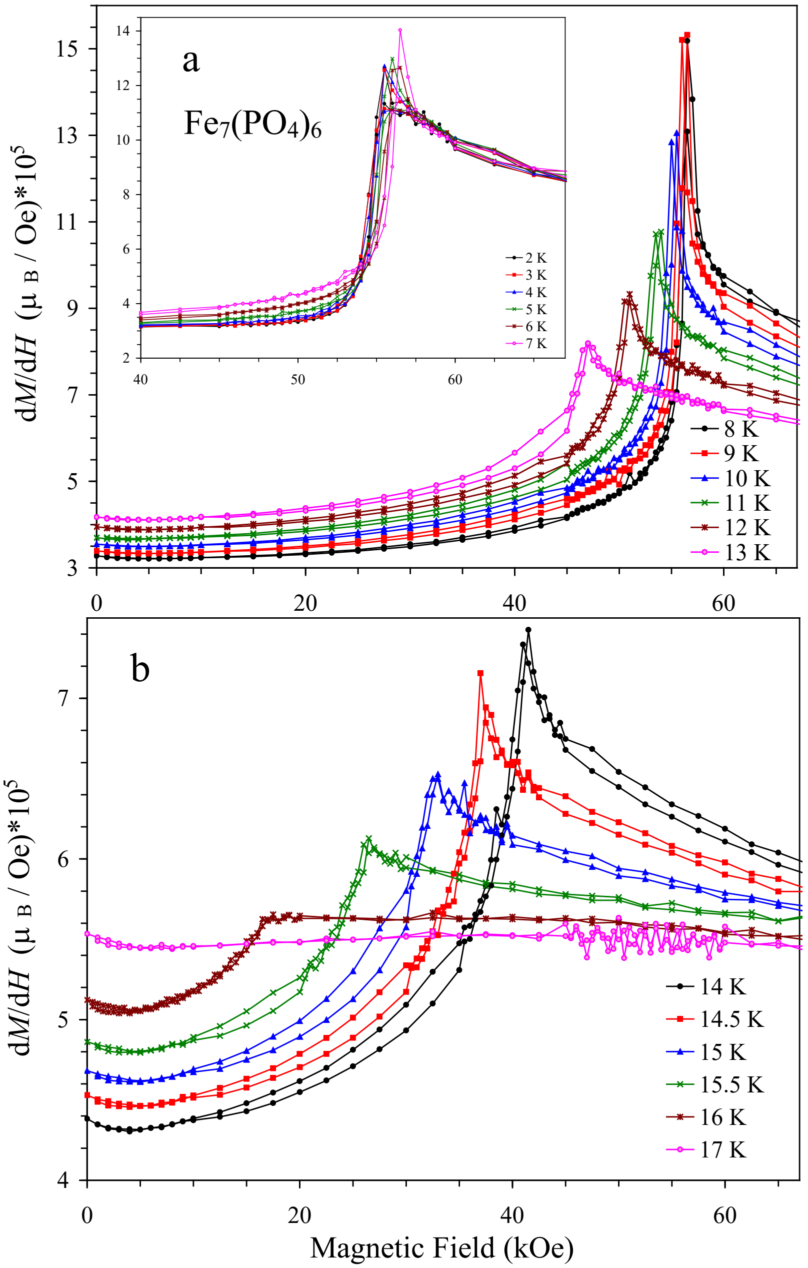

Figure S6. (a) and (b) Differential d*M*/d*H* versus *H* curves (up and down) at different temperatures for $Fe_7(PO_4)_6$. Inset in (a) shows the zoomed-in part at *T* = 2, 3, 4, 5, 6, and 7 K.

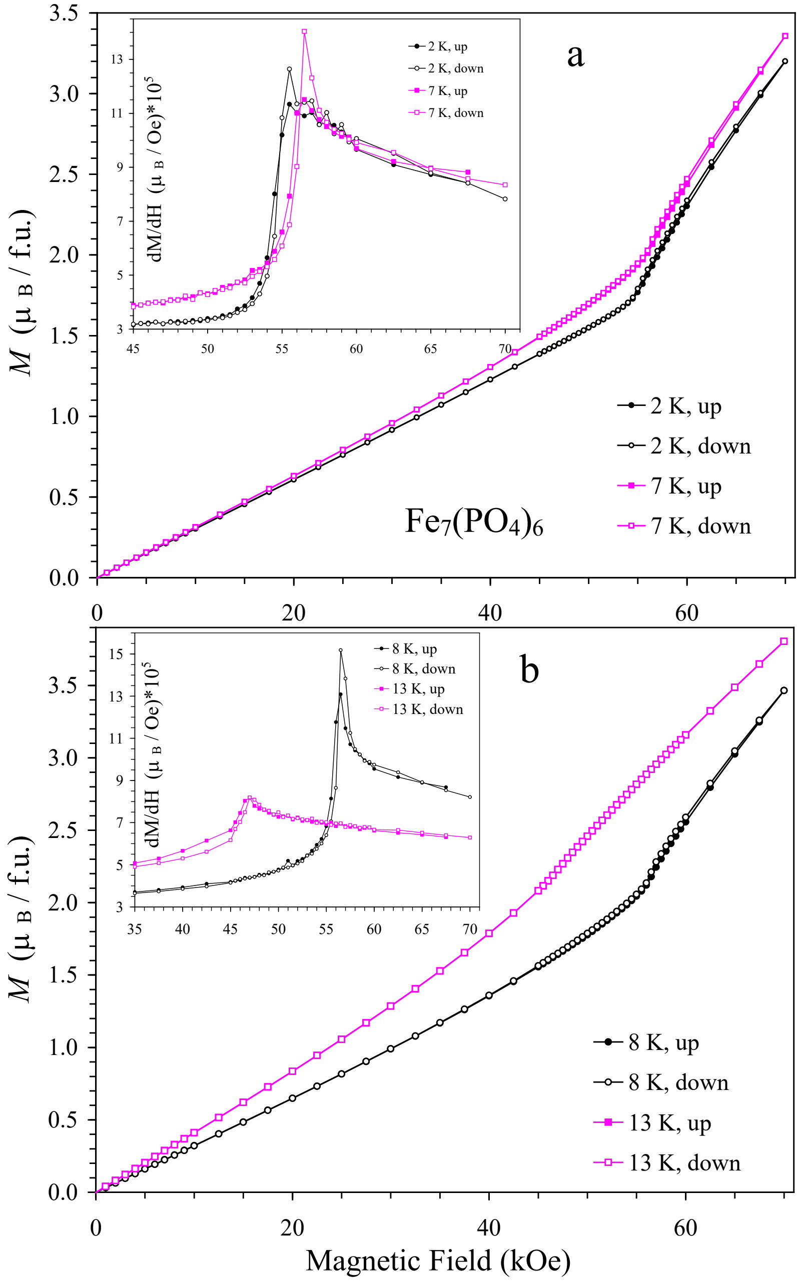

Figure S7. *M* versus *H* curves at some selected temperatures of (a) $T = 2$ K and 7 K and (b) $T = 8$ K and 13 K measured from 0 Oe to 70 kOe (up) and from 70 kOe to 0 Oe (down) for $Fe_7(PO_4)_6$. Insets show the zoomed-in differential d*M*/d*H* versus *H* curves.

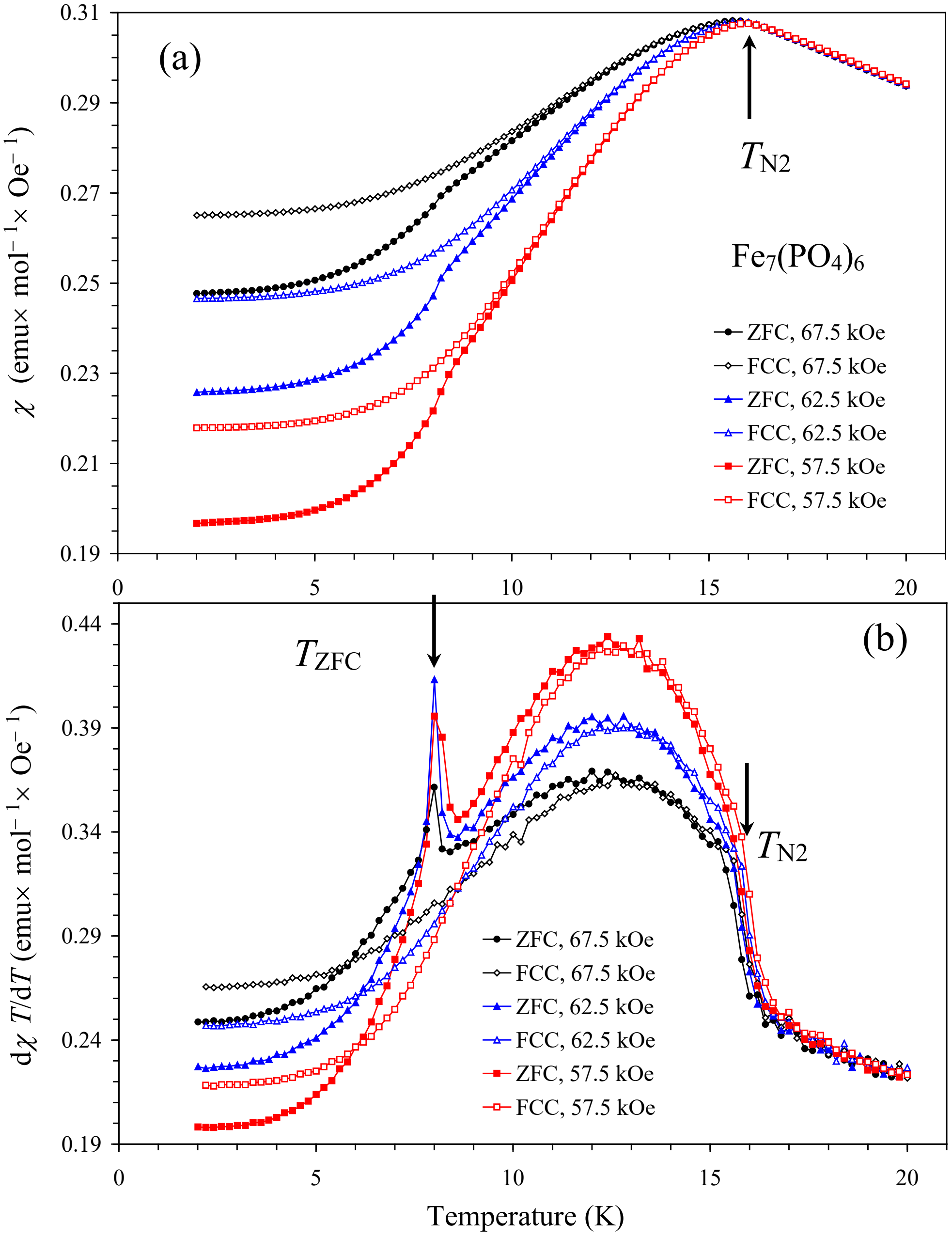

Figure S8. (a) χ versus T curves at H = 67.5, 62.5, and 57.5 kOe measured in the ZFC and FCC (on cooling) regimes for $Fe_7(PO_4)_6$. (b) The same differential dχT/dT versus T curves. Arrows show the magnetic anomalies.

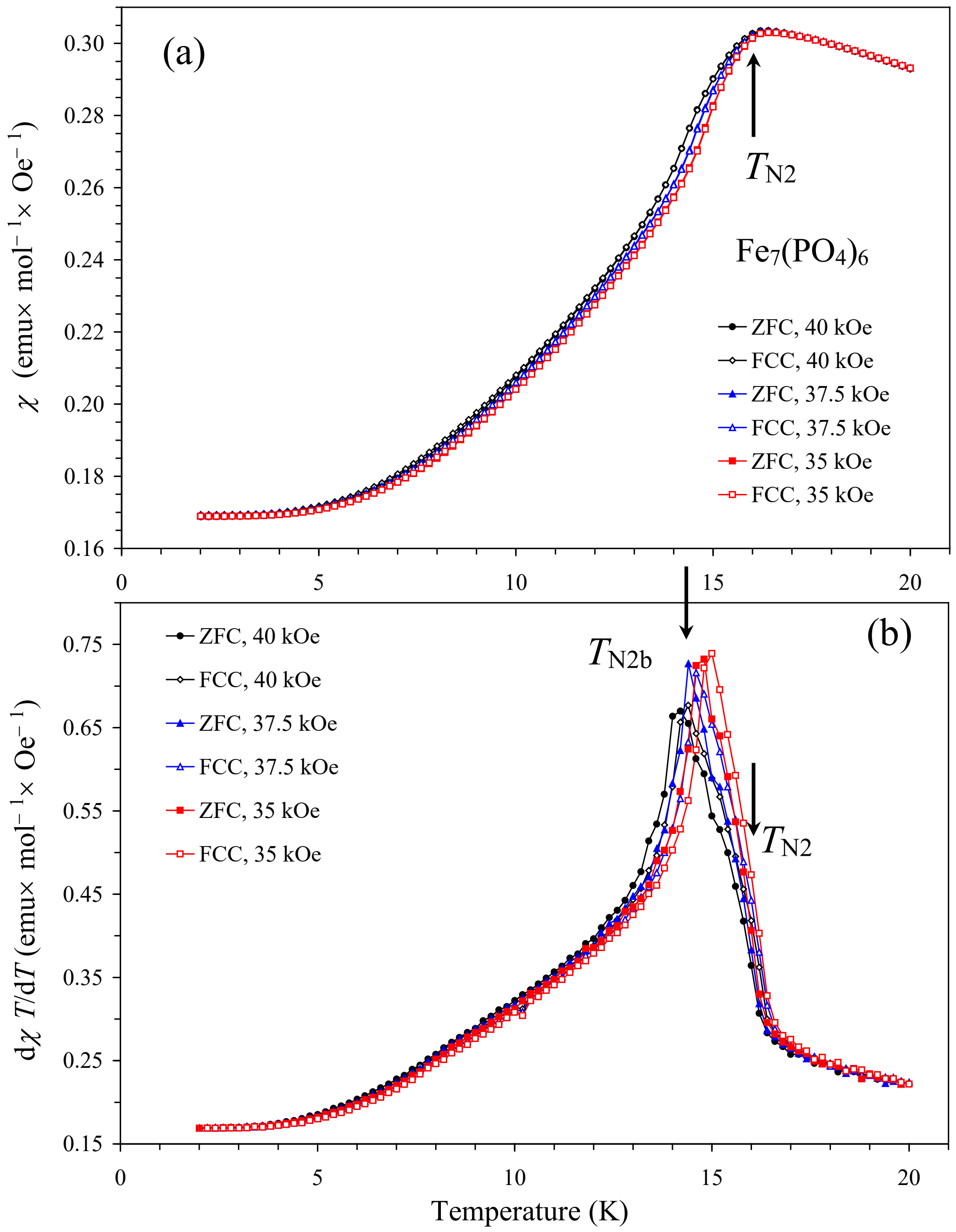

Figure S9. (a) χ versus T curves at H = 40.0, 37.5, and 35.0 kOe measured in the ZFC and FCC (on cooling) regimes for $Fe_7(PO_4)_6$. (b) The same differential dχT/dT versus T curves. Arrows show the magnetic anomalies.

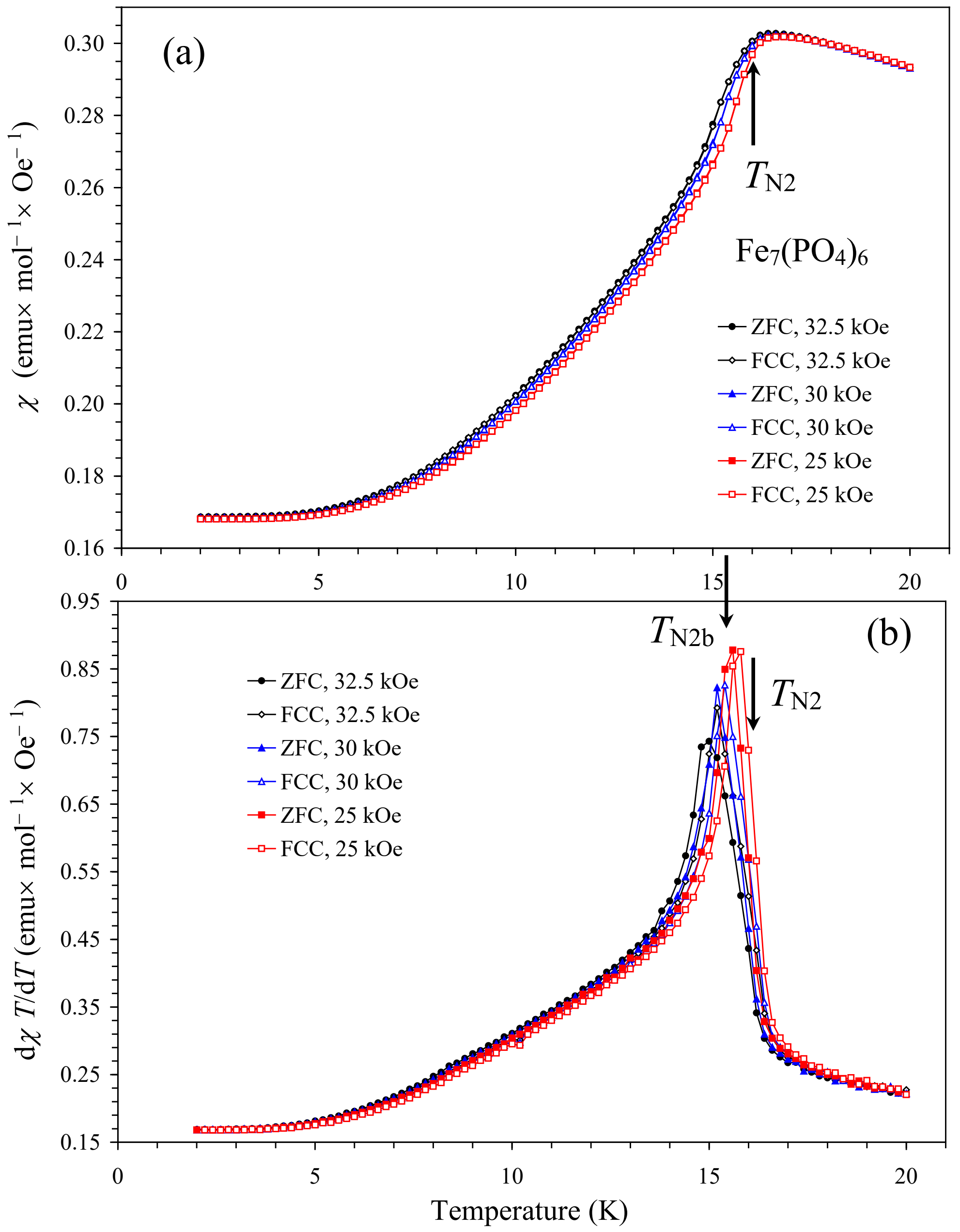

Figure S10. (a) χ versus T curves at H = 32.5, 30.0, and 25.0 kOe measured in the ZFC and FCC (on cooling) regimes for $Fe_7(PO_4)_6$. (b) The same differential dχT/dT versus T curves. Arrows show the magnetic anomalies.

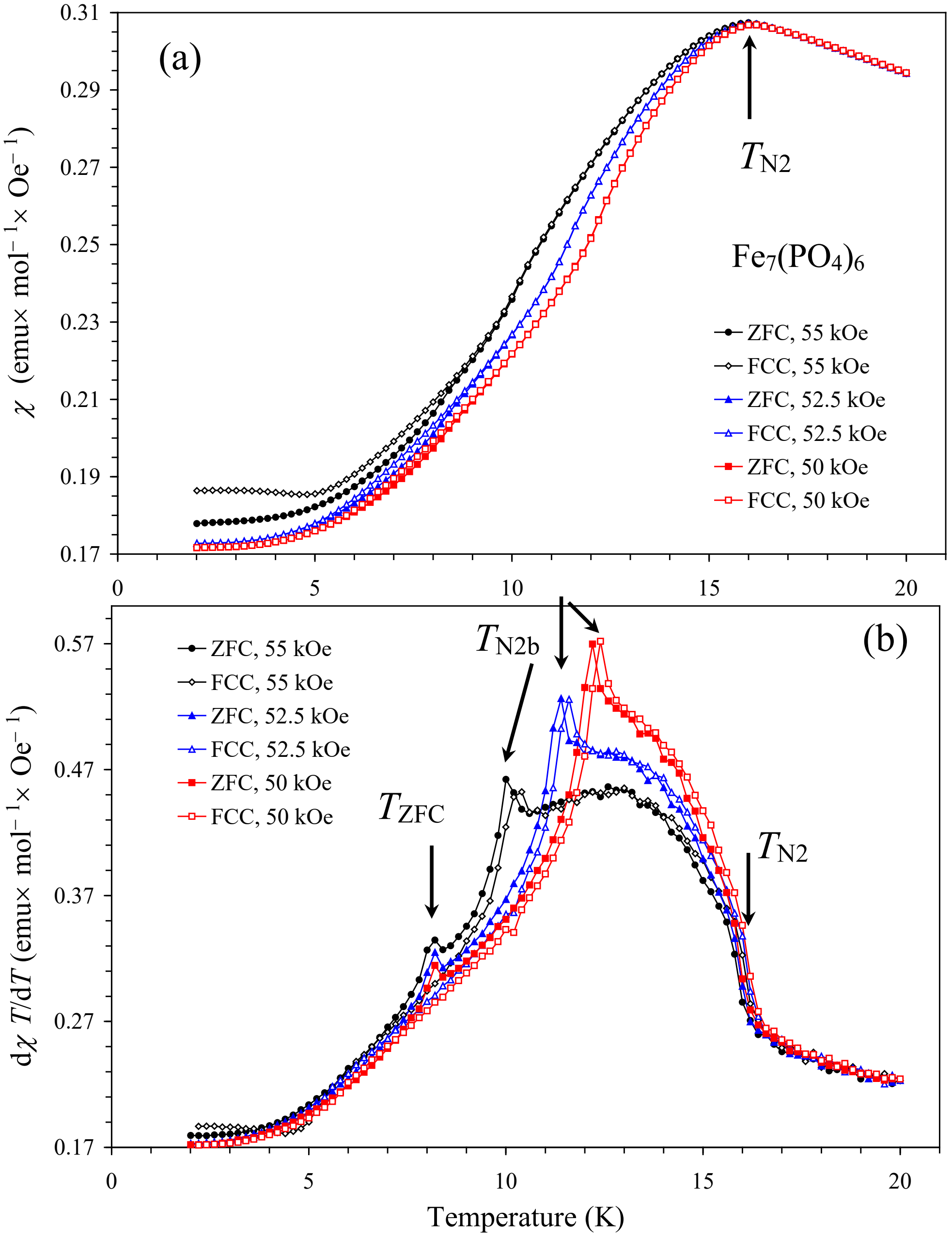

Figure S11. (a) χ versus T curves at H = 55.0, 52.5, and 50.0 kOe measured in the ZFC and FCC (on cooling) regimes for $Fe_7(PO_4)_6$. (b) The same differential $d\chi T/dT$ versus T curves. Arrows show the magnetic anomalies.

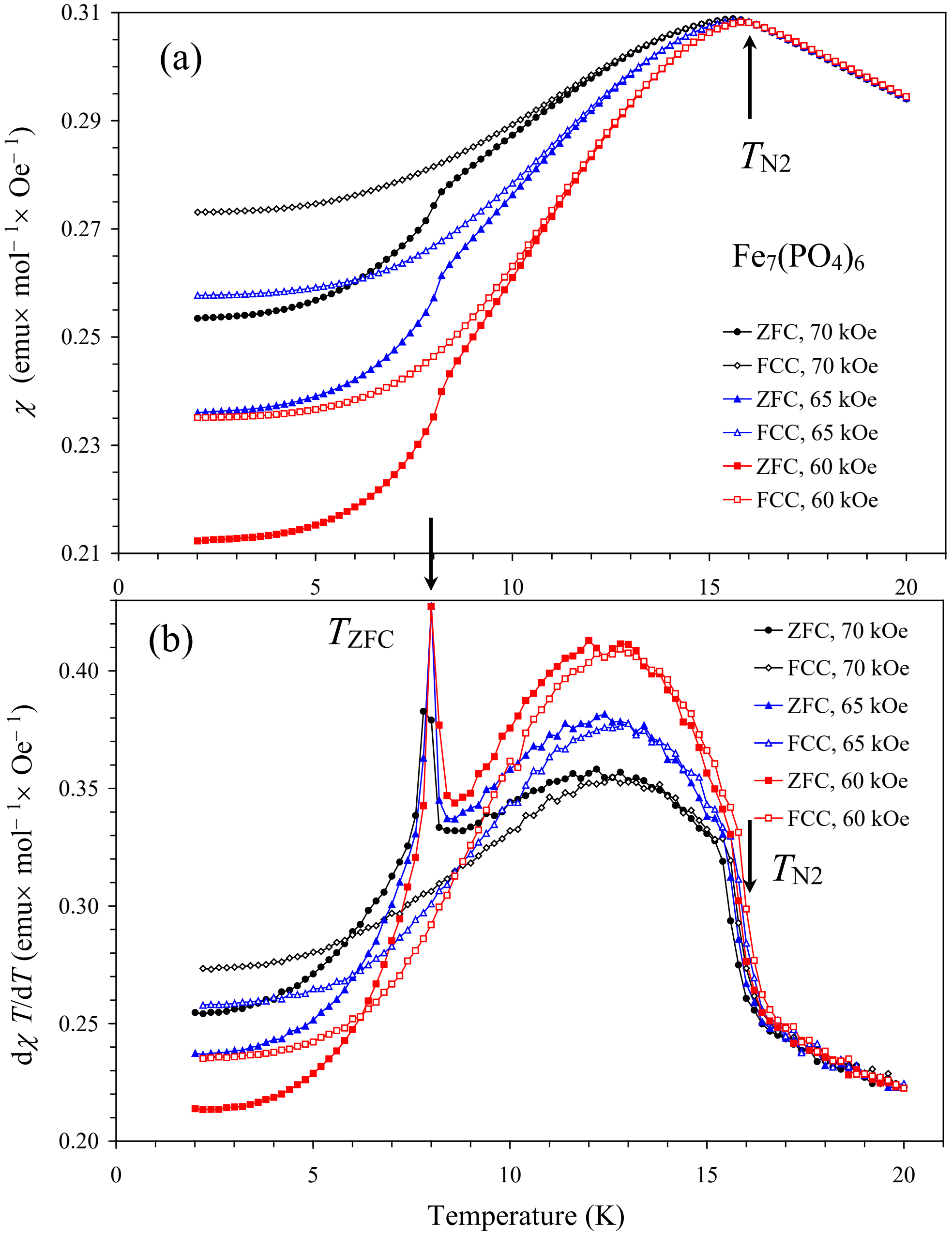

Figure S12. (a) χ versus T curves at H = 70.0, 65.0, and 60.0 kOe measured in the ZFC and FCC (on cooling) regimes for $Fe_7(PO_4)_6$. (b) The same differential $d\chi T/dT$ versus T curves. Arrows show the magnetic anomalies.

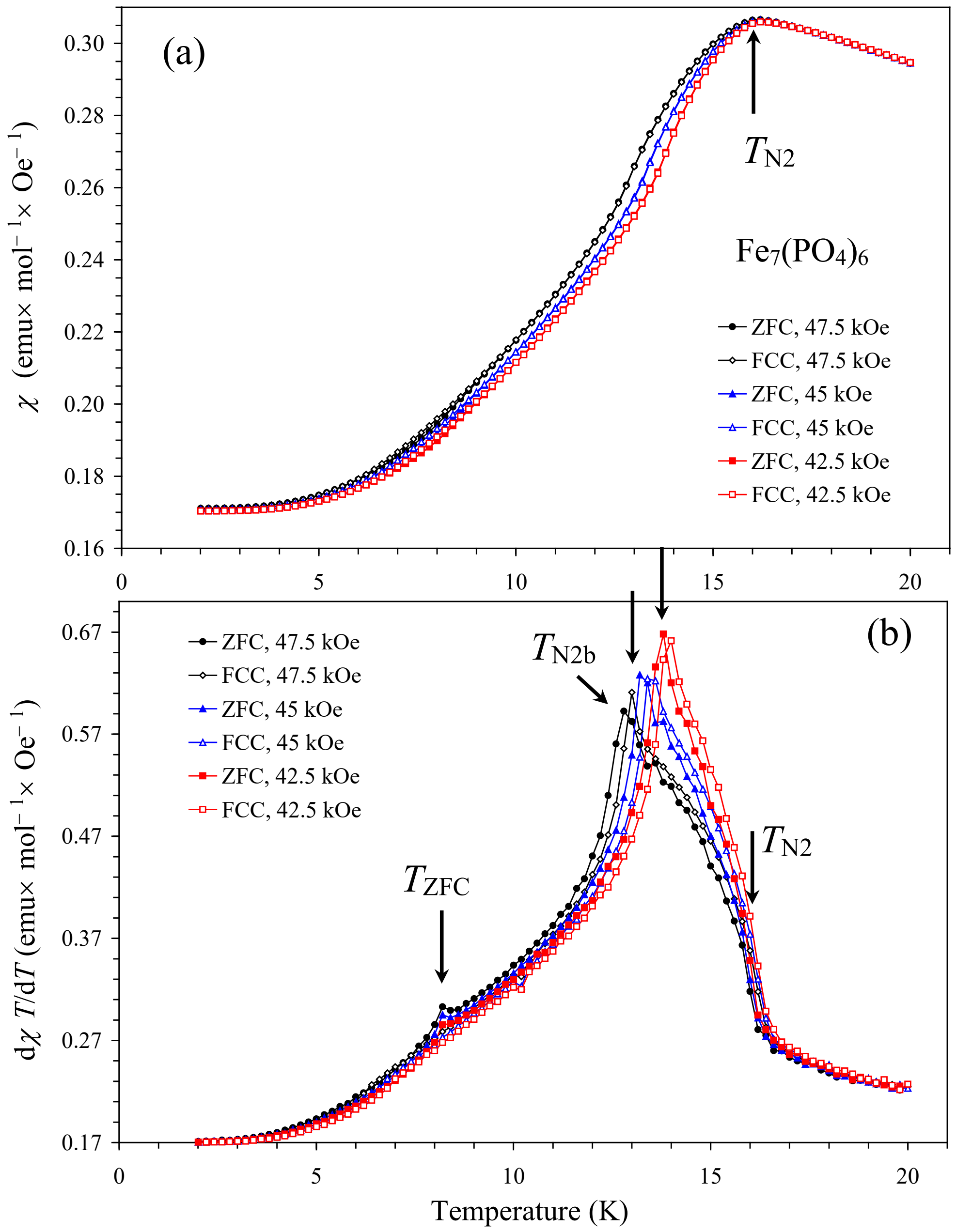

Figure S13. (a) χ versus T curves at H = 47.5, 45.0, and 42.5 kOe measured in the ZFC and FCC (on cooling) regimes for $Fe_7(PO_4)_6$. (b) The same differential dχT/dT versus T curves. Arrows show the magnetic anomalies.

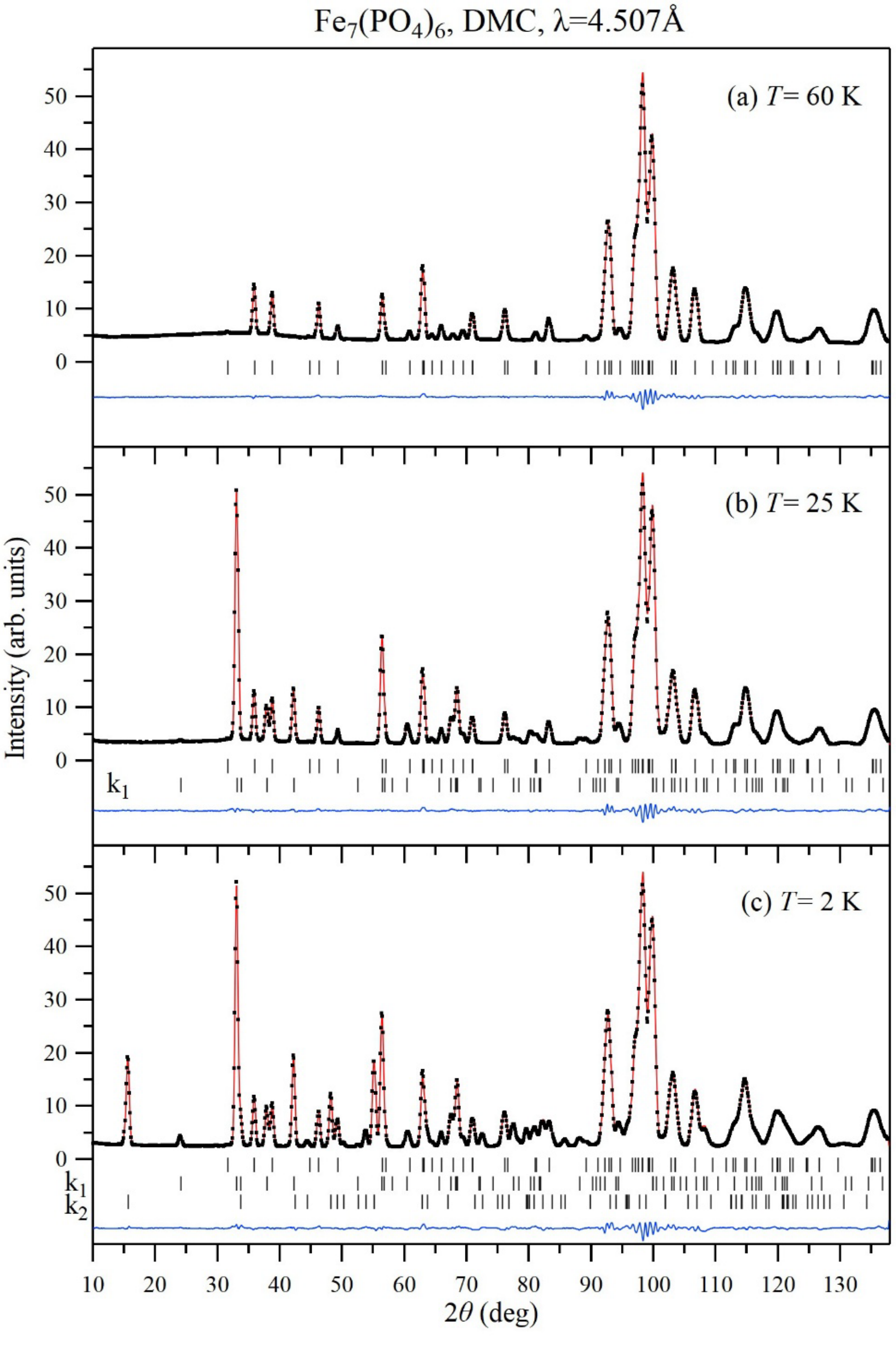

Figure S14. Experimental (black dots), calculated (red line), and difference (blue line) neutron diffraction patterns of $Fe_7(PO_4)_6$ measured on DMC with a neutron wavelength λ = 4.507 Å in the paramagnetic state at T = 60 K (a) and in the magnetically ordered states at T = 25 K (b), and 1.8 K (c). Tick marks indicate Bragg peak positions. The first row is for the nuclear peaks, and the second and third rows are for the magnetic peaks.

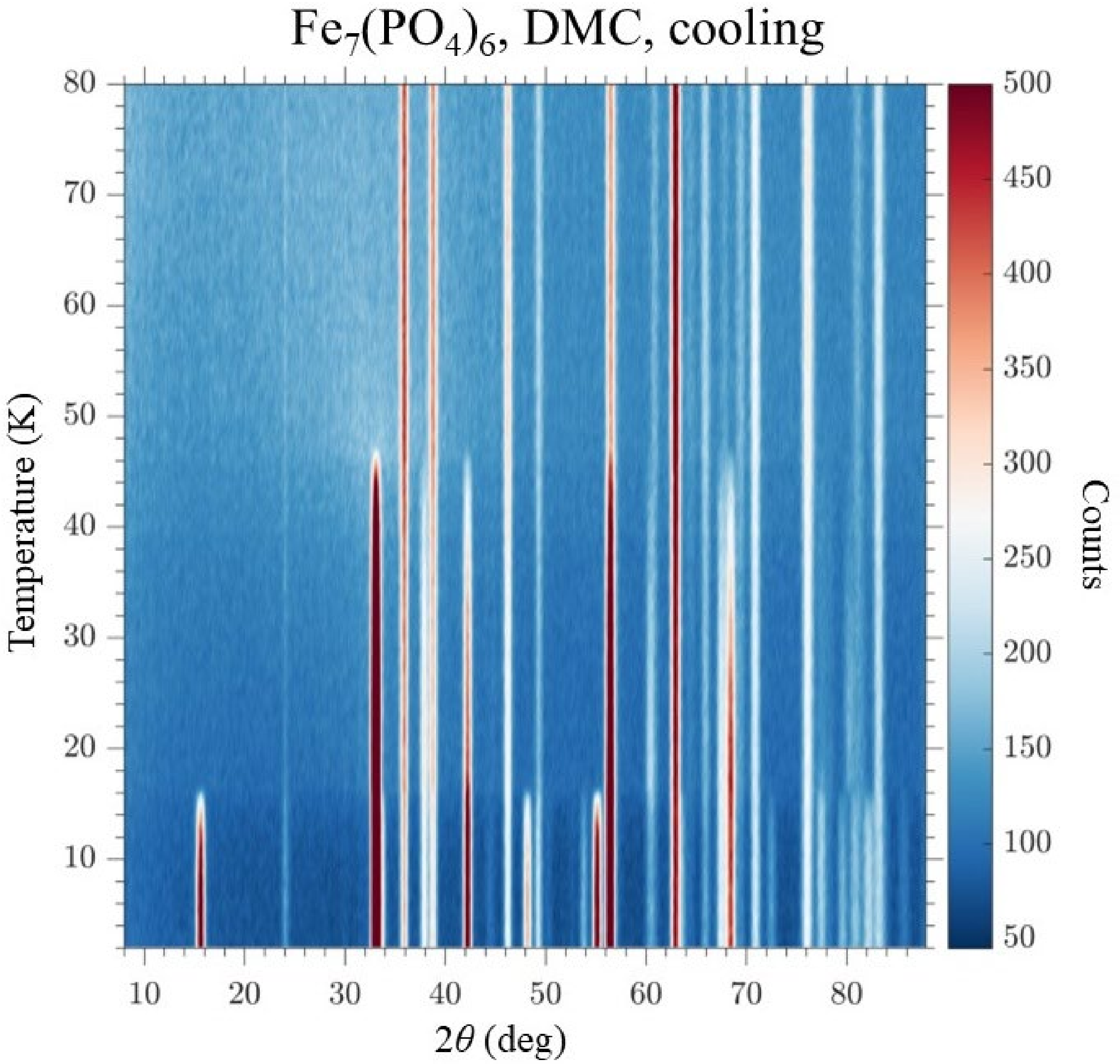

Figure S15. Temperature and 2θ dependent neutron intensity map of $Fe_7(PO_4)_6$ measured on DMC for cooling from 80 K.

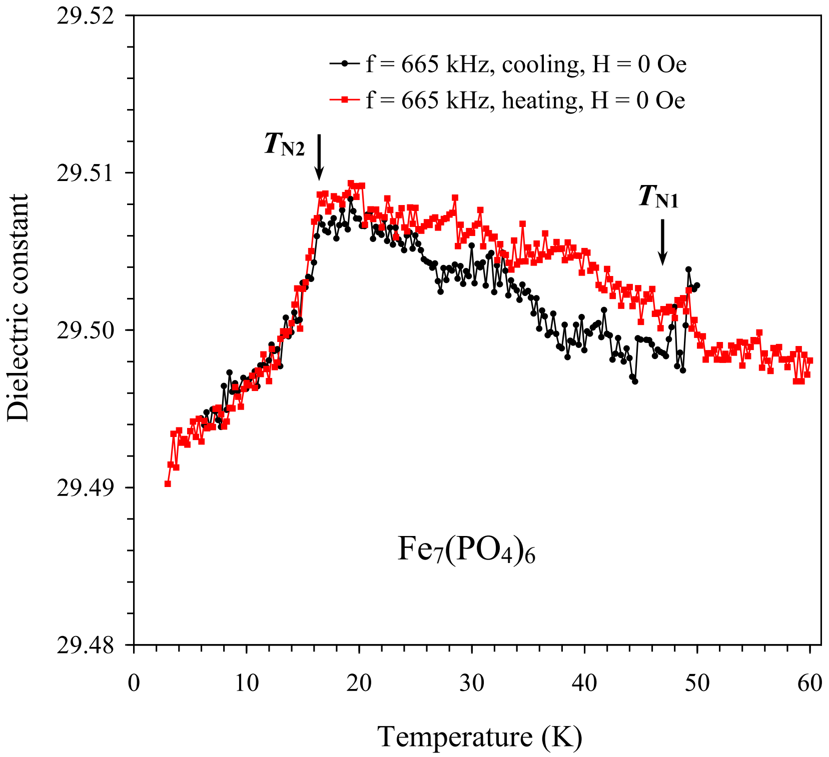

Figure S16. Temperature-dependent dielectric curves of $Fe_7(PO_4)_6$ on cooling (black) and heating (red) measured at $H = 0$ Oe. Data at one frequency of 665 kHz are shown for clarity. There were no anomalies at T_{N1}, while there were small kink-like anomalies at T_{N2}, suggesting a small magnetostriction effect. These dielectric data also confirm insulating properties. Dielectric properties were measured using an Alpha-A High Performance Frequency Analyzer (NOVOCONTROL Technologies, Montabaur, Germany) on cooling and heating.

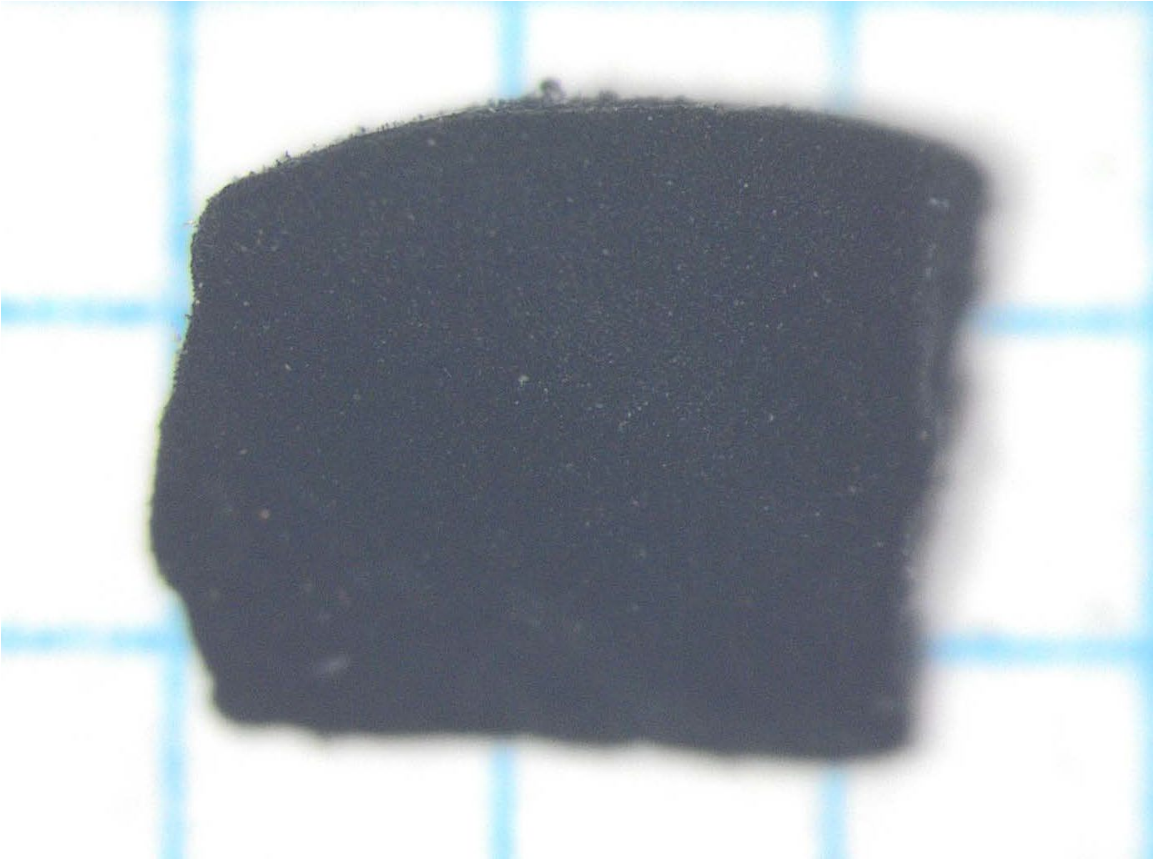

Figure S17. A photo of a pellet of $Fe_7(PO_4)_6$ (6.75 mg) used for specific heat measurements. Distance between the blue lines is 1 mm.
